# Single-cell transcriptomics identifies PDGFRA^+^ progenitors orchestrating angiogenesis and periodontal tissue regeneration

**DOI:** 10.1038/s41368-025-00384-6

**Published:** 2025-07-24

**Authors:** Jianing Liu, Junxi He, Ziqi Zhang, Lu Liu, Yuan Cao, Xiaohui Zhang, Xinyue Cai, Xinyan Luo, Xiao Lei, Nan Zhang, Hao Wang, Ji Chen, Peisheng Liu, Jiongyi Tian, Jiexi Liu, Yuru Gao, Haokun Xu, Chao Ma, Shengfeng Bai, Yubohan Zhang, Yan Jin, Chenxi Zheng, Bingdong Sui, Fang Jin

**Affiliations:** 1 State Key Laboratory of Oral & Maxillofacial Reconstruction and Regeneration, National Clinical Research Center for Oral Diseases, Shaanxi International Joint Research Center for Oral Diseases, Center for Tissue Engineering, School of Stomatology, The Fourth Military Medical University, Xi’an, China; 2 State Key Laboratory of Oral & Maxillofacial Reconstruction and Regeneration, National Clinical Research Center for Oral Diseases, Shaanxi Clinical Research Center for Oral Diseases, Department of Orthodontics, School of Stomatology, The Fourth Military Medical University, Xi’an, China; 3 State Key Laboratory of Oral & Maxillofacial Reconstruction and Regeneration, National Clinical Research Center for Oral Diseases, Shaanxi Clinical Research Center for Oral Diseases, Department of Preventive Dentistry, School of Stomatology, The Fourth Military Medical University, Xi’an, China; 4 State Key Laboratory of Oral & Maxillofacial Reconstruction and Regeneration, National Clinical Research Center for Oral Diseases, Shaanxi Engineering Research Center for Dental Materials and Advanced Manufacture, Department of Oral Implantology, School of Stomatology, The Fourth Military Medical University, Xi’an, China

**Keywords:** Mesenchymal stem cells, Regeneration

## Abstract

Periodontal bone defects, primarily caused by periodontitis, are highly prevalent in clinical settings and manifest as bone fenestration, dehiscence, or attachment loss, presenting a significant challenge to oral health. In regenerative medicine, harnessing developmental principles for tissue repair offers promising therapeutic potential. Of particular interest is the condensation of progenitor cells, an essential event in organogenesis that has inspired clinically effective cell aggregation approaches in dental regeneration. However, the precise cellular coordination mechanisms during condensation and regeneration remain elusive. Here, taking the tooth as a model organ, we employed single-cell RNA sequencing to dissect the cellular composition and heterogeneity of human dental follicle and dental papilla, revealing a distinct Platelet-derived growth factor receptor alpha (PDGFRA) mesenchymal stem/stromal cell (MSC) population with remarkable odontogenic potential. Interestingly, a reciprocal paracrine interaction between PDGFRA^+^ dental follicle stem cells (DFSCs) and CD31^+^ Endomucin^+^ endothelial cells (ECs) was mediated by Vascular endothelial growth factor A (VEGFA) and Platelet-derived growth factor subunit BB (PDGFBB). This crosstalk not only maintains the functionality of PDGFRA^+^ DFSCs but also drives specialized angiogenesis. In vivo periodontal bone regeneration experiments further reveal that communication between PDGFRA^+^ DFSC aggregates and recipient ECs is essential for effective angiogenic-osteogenic coupling and rapid tissue repair. Collectively, our results unravel the importance of MSC-EC crosstalk mediated by the VEGFA and PDGFBB-PDGFRA reciprocal signaling in orchestrating angiogenesis and osteogenesis. These findings not only establish a framework for deciphering and promoting periodontal bone regeneration in potential clinical applications but also offer insights for future therapeutic strategies in dental or broader regenerative medicine.

## Introduction

Periodontal bone defects occur in excessive orthodontic tooth movement and periodontitis with high prevalence, manifesting as bone fenestration, dehiscence or attachment loss, which impose a challenging issue in oral health.^1,2^ Over the last two decades, the rapid growth of stem cell research with the concerted emergence of multiple biotechnologies has remarkably led to various approaches toward tissue and organ regeneration.^3–5^ Particularly, a promising framework yet being a critical challenge for both biology and regenerative medicine is to harness the organizational and communicational principles in development that generate complex tissue topography and govern organ-level functionality.^6,7^ Stem cells, especially mesenchymal stem/stromal cells (MSCs), possess an intrinsic property to form compact and integrated cell assemblies, a phenomenon known as cell condensation/aggregation.^8,9^ Researchers have demonstrated that dissociating condensed tooth germs into single cells and subsequently reaggregating the epithelial and mesenchymal components can lead to the regeneration of tooth structures through epithelial-mesenchymal induction, a process that precisely exploits the principle of condensation.^10^ Recently, mesenchymal condensation has been revealed as an essential event for initiating organogenesis in a variety of organs, such as the tooth as a representative model organ.^8,9^ Accordingly, we have established a development-inspired biomimetic strategy to construct engineered MSC aggregates and successfully introduced it into clinical practice for human dental pulp and periodontal regeneration.^11–13^ Recent studies have significantly advanced our understanding of MSC heterogeneity, revealing substantial variations in differentiation potential at the single-cell level.^14^ In the context of tooth development, studies in mouse models have identified discrete mesenchymal subpopulations that constitute the dental mesenchyme at different developmental phases.^15,16^ Particularly noteworthy are CD24a^+^ multipotent dental pulp stem cells, which demonstrate exceptional odontogenic and osteogenic capacity under three-dimensional (3D) culture conditions, positioning them as prime candidates for regenerative therapies targeting pulpitis and pulp necrosis.^17^ However, critical knowledge gaps remain regarding the precise identity of key stem cell populations orchestrating organogenetic condensation, their dynamic interactions with niche components, and strategies to enhance the therapeutic efficacy of MSC aggregates within recipient microenvironments for clinical translation.^18^ Therefore, deciphering in-depth the cellular composition of mesenchymal condensation and the mechanisms underlying its contribution to tissue development will be beneficial to guide efficient translational regeneration.

In organ development, mesenchymal condensation not only dictates subsequent tissue formation and patterning through cellular self-organization but also acts as the signaling niche to orchestrate specification of interlineage progenitors.^8,9,19^ Specifically, the cell lineage communication based on mesenchymal aggregation during organogenesis involves the regulation of angiogenesis in organ bud formation.^20^ Taking odontogenesis as an example, the primordial mesenchymal condensation is induced by the dental epithelium in an initial environment devoid of vascularization,^21^ which then recruits endothelial progenitor cells (EPCs) and promotes their assembly into a primary vascular network.^22,23^ Concurrently, the primordial mesenchymal condensation undergoes differentiation to form the dental papilla and follicle condensations, which develop respectively into the tooth pulp and periodontium.^16,24^ Interestingly, both the dental papilla and the dental follicle tissues continue to exist postnatally in humans despite being restricted to specific regions of unerupted or immature teeth, which can be safely harvested for isolation and regenerative application of dental MSCs, known as stem cells from the apical papilla (SCAP) and dental follicle stem cells (DFSCs).^25,26^ However, whether these dental MSCs interplay with EPCs or endothelial cells (ECs) to coordinate angiogenesis with odontogenesis during embryonic and postnatal development remains elusive. We have further reported that MSC aggregates-facilitated human tissue regeneration involves vascular reconstruction,^12,13^ and that angiogenesis couples with tissue formation in multiple regenerative conditions, notably regarding a CD31/Platelet endothelial cell adhesion molecule 1 (PECAM1)- and Endomucin (EMCN)-highly co-expressed vessel subtype.^27,28^ Nevertheless, the precise mechanisms by which specific MSC-EC populations interact to safeguard tissue regeneration warrant further investigations.

In this study, we aimed to establish an optimized paradigm from dissecting mesenchymal condensation-mediated tissue development to promoting MSC aggregates-facilitated tissue regeneration by focusing on detailed stem cell contribution and interlineage cell crosstalk in the odontogenic microenvironment. To tackle this complex issue, we employed single-cell RNA sequencing (scRNA-seq) to delve into the cellular composition and heterogeneity within the dental follicle and dental papilla developing tissues. Our scRNA-seq analyses, combined with cellular and molecular experimental efforts, discover a reciprocal paracrine signaling mechanism between Platelet-derived growth factor receptor alpha (PDGFRA)^+^ MSCs and EPCs/ECs mediated by Vascular endothelial growth factor A (VEGFA) and Platelet-derived growth factor subunit BB (PDGFBB). Given the purpose of addressing the issue of periodontal bone defects, and considering that DFSCs are isolated and cultured from dental follicle tissue, the origin of periodontal tissues, we make a commitment to leverage the developmental principle to benefit the periodontal.^29,30^ Interestingly, the interaction supports DFSC functionality with the formation of CD31^+^EMCN^+^ vessels in odontogenesis and promotes periodontal regeneration. Collectively, our results unravel a specialized mesenchymal-endothelial interplay in tissue development and regeneration related to odontogenic condensation. These findings will add to the knowledge of development-inspired periodontal regeneration and be beneficial to establishing feasible translational strategies for clinical tissue repair.

## Results

### Single-cell transcriptomic profiling of human dental developing tissues identifies characteristic stem cell populations

**Fig. 1:**
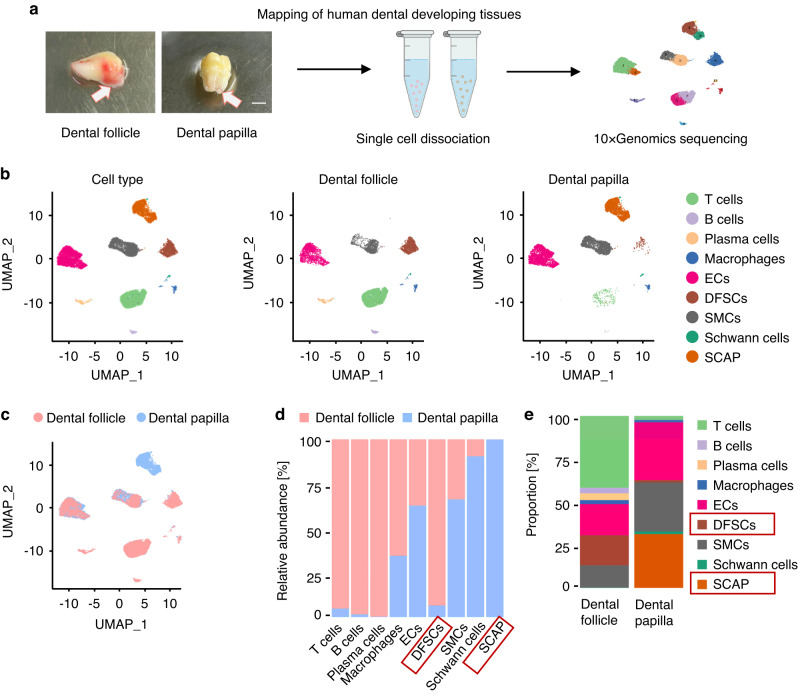
Single-cell transcriptomic profiling of human dental developing tissues identifies characteristic stem cell populations. **a** Schematic illustration showing the experimental workflow from human dental tissue preparation to a mapping of major cell clusters. **b** UMAP plots of 9 947 cells from the dental follicle and 9 048 cells from the dental papilla, colored by nine cell-type annotations. **c** UMAP plot of cluster distribution in the merged dental follicle and dental papilla data set. **d** Bar plot of the relative abundance of different cell types in dental follicle and dental papilla tissues. **e** The proportion of clusters in dental follicle and dental papilla tissues

To begin with, we dissected the characteristics of representative postnatal human developing dental tissues, the dental follicle and the dental papilla (Fig. 1a). Impacted mandibular third molars were collected, and single-cell transcriptomic data encompassing 9 997 cells from the dental follicle and 9 048 cells from the dental papilla were generated using the 10x Genomics scRNA-seq technology (Fig. 1a). Cells were categorized into 9 distinct clusters by specific markers (Fig. 1b), including DFSCs (*Periostin*, *POSTN*, and *Secreted protein acidic and cysteine rich*, *SPARC*), SCAP (*Pleiotrophin*, *PTN*, and *WNT inhibitory factor 1*, *WIF1*), ECs (*PECAM1*/*CD31* and *EMCN*), Schwann cells (*GDNF family receptor alpha 3*, *GFRA3*, and *S100 calcium binding protein B*, *S100B*), smooth muscle cells (SMCs) (*Actin alpha 2*, *ACTA2*, and *Myosin heavy chain 11*, *MYH11*), T cells (*CD3E* and *CD3D*), B cells (*CD19* and *CD79B*), plasma cells (*X-box binding protein 1*, *XBP1*, and *CD38*), and macrophages (*Complement C1q A chain*, *C1QA*, and *Allograft inflammatory factor 1*, *AIF1*) (Fig. S1a). Comparative analysis on the relative abundance of different cell populations in the two dental tissue datasets revealed a significant enrichment of DFSCs and multiple immune cells in the dental follicle, while the dental papilla demonstrated enrichment of SCAP with Schwann cells, ECs, and SMCs (Fig. 1c, d). Indeed, both tissues harbored a remarkable stem cell population (DFSCs and SCAP) with abundant ECs and SMCs, whereas the dental follicle particularly contained a high proportion of T cells (Fig. 1e). Hematoxylin and eosin (H&E) staining results of the dental follicle and dental papilla also confirmed that there were more conspicuous lymphocytes infiltrated in the dental follicle than the dental papilla (Fig. S2). These intriguing findings suggest potentially common stem, endothelial, and mesenchymal features of the two tissues, with differences mainly in the immune microenvironmental involvement.

We continued to analyze the stem cell populations in the dental follicle and the dental papilla. ScRNA-seq data further demonstrated the heterogeneity of stem cells in both tissues, with DFSCs being divided into five subclusters and SCAP into four subclusters (Fig. S3a, b). The specific functions of these subclusters were inferred from their molecular signatures. For example, the DFSC subcluster marked by *Integrin subunit alpha 8* (*ITGA8*) and the SCAP subcluster marked by *Sphingosine kinase 1* (*SPHK1*) existed at the initiation of respective developmental trajectories and were critical for periodontal or dental pulp tissue formation (Figs. S1b and S3c, d). In vitro culture validated the MSC identity of both DFSCs and SCAP based on the presence of CD90 and the absence of CD45 surface antigens, colony-forming capabilities, and the osteogenic differentiation potential (Fig. S4a–c). Taken together, these results identify characteristic stem cell populations in human dental developing tissues.

### PDGFRA hallmarks common dental progenitor cells in DFSCs and SCAP in situ

**Fig. 2:**
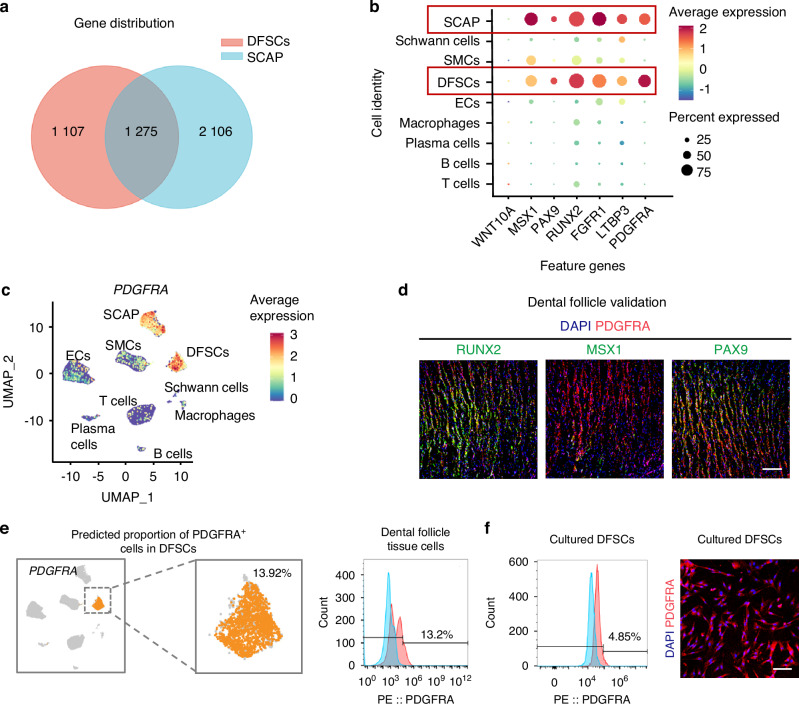
PDGFRA hallmarks common dental progenitor cells in DFSCs and SCAP in situ. **a** Venn diagram showing gene distribution of DFSCs and SCAP with 1275 co-expressed genes. **b** Bubble plot showing the expression of typical genes associated with odontogenesis in dental follicle and papilla cell clusters. **c** Feature plot showing *PDGFRA* expression distribution in the merged dental follicle and dental papilla data set. **d** IF staining images showing RUNX2 (green), MSX1 (green), or PAX9 (green) co-localized with PDGFRA (red) in the dental follicle tissue. Scale bar = 200 μm. **e** Predicted proportion of PDGFRA^+^ cells in DFSCs and flow cytometric analysis for the proportion of PDGFRA^+^ cells in dissociated dental follicle tissue cells. **f** Flow cytometric analysis for the proportion of PDGFRA^+^ cells in cultured DFSCs and IF staining images showing PDGFRA^+^ (red) cells in cultured DFSCs. Scale bar = 100 μm

Next, we investigated whether DFSCs and SCAP, with similar origins and functionality, may possess common molecular features. An in-depth analysis of the scRNA-seq data revealed genes specifically expressed in DFSCs and SCAP, while a total of 1 275 genes were found to be co-expressed in both stem cell types (Fig. 2a). Notably, genes relevant to the odontogenesis process were dramatically highly expressed in both DFSCs and SCAP over other cell types in the dental follicle and papilla tissues, such as the transcription factor genes, *Msh homeobox 1* (*MSX1*), *Paired box 9* (*PAX9*), and *Runt-related transcription factor 2* (*RUNX2*), and the growth factor signaling genes, *Fibroblast growth factor receptor 1* (*FGFR1*), *Latent transforming growth factor beta binding protein 3* (*LTBP3*) (Fig. 2b). Importantly, to identify genes that are commonly highly expressed in both DFSCs and SCAP to mark dental progenitor cells, a combined analysis of differentially expressed genes (DEGs) of these two types of MSCs compared with other cell populations was performed. It was found that PDGFRA was the only membrane surface protein related to mesodermal/mesenchymal tissue development that ranked within the top 15 of DEGs (Table S1). Also, other markers possibly served as indicators of dental progenitor cells, even though some were not expressed on the cell membrane, including *Prostaglandin F2-alpha receptor* (*PTGFR*), *Multiple epidermal growth factor-like-domains 10* (*MEGF10*), *Type II deiodinase* (*DIO2*), and *FGFR2* (Table S1). This provides an important reference for interpreting the characteristics of dental progenitor cells. Particularly, PDGFRA is an established surface marker of MSCs,^31^ which has recently been unraveled to play a critical role in tooth formation.^32^ As expected, *PDGFRA* showed expression specificity in DFSCs and SCAP (Fig. 2c). Immunofluorescence (IF) staining of PDGFRA confirmed their preferential expression in RUNX2-, MSX1-, and PAX9-labeled odontogenesis-potent cells both in the dental follicle and the dental papilla tissues (Figs. 2d and S5a). The proportion of PDGFRA^+^ cells in the dental follicle tissue was further validated by flow cytometry, consistent with theoretical predictions of 13%–14% based on bioinformatic analysis (Fig. 2e). Those cells were still detected after in vitro culture of DFSCs, although at a percentage of less than 5% (Fig. 2f). The expression of PDGFRA was also confirmed by in vitro culture of SCAP (Fig. S5b). Together, these findings indicate that PDGFRA hallmarks common dental progenitor cells in DFSCs and SCAP in situ.

### PDGFRA^+^ DFSCs do not show functional superiority over PDGFRA^−^ DFSCs in vitro

**Fig. 3:**
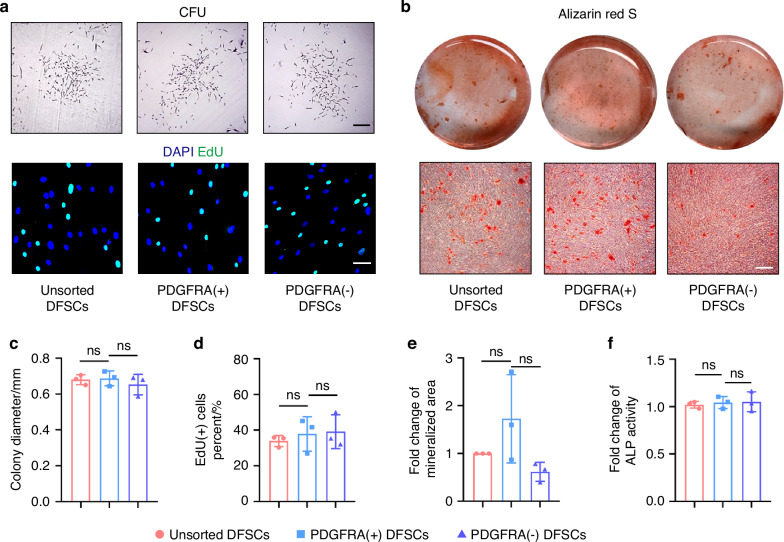
PDGFRA^+^ DFSCs do not show functional superiority over PDGFRA^−^ DFSCs in vitro. **a** Representative images of colonies formed by DFSCs (scale bar = 250 μm) and EdU staining images showing DFSC proliferation (scale bar = 50 μm). **b** Alizarin red S staining images showing DFSC osteogenesis. Scale bar = 100 μm. **c** Quantification of colony diameter in CFU assay. **d** Quantification of EdU^+^ cells percent (%). **e** Quantification of fold change of mineralized area. **f** Quantification of the fold change of ALP activity. Data were presented as mean ± SD. *n* = 3 per group. ns not significant (*P* > 0.05)

The above results inspired us to investigate whether PDGFRA^+^ dental progenitor cells have advantages in stem cell function. The dental follicle is the developmental origin of periodontal tissues and forms the alveolar bone, the periodontal ligament, and the cementum during tooth development.^29,30^ The colony formation assay further demonstrated that DFSCs have stronger proliferation capacity, osteogenic differentiation potential, and the ability to induce EC tube formation compared to SCAP (Fig. S4b–d). Therefore, we selected DFSCs as further candidates for functional assays, aiming to find optimized seed cell populations for periodontal regeneration. We employed the magnetic cell sorting technique to separate PDGFRA^+^ DFSCs from their negatively expressed counterparts (Fig. S6a, b). Surprisingly, no significant difference in the self-renewal capacity was observed among unsorted DFSCs, PDGFRA^+^ DFSCs, and PDGFRA^−^ DFSCs, as assessed by the colony-forming unit (CFU) assay (Fig. 3a). Furthermore, labeling of DNA replication-competent cells by 5-ethynyl-2′-deoxyuridine (EdU) demonstrated no difference in the proliferation index among groups (Fig. 3a). Moreover, alizarin red S staining of mineralization after osteogenic induction of DFSCs did not show a difference among groups (Fig. 3b). Statistical analyses confirmed these findings, and examination of alkaline phosphatase (ALP) activity verified the comparable osteogenesis of DFSCs irrespective of the PDGFRA status (Fig. 3c–f). Additionally, quantitative real-time polymerase chain reaction (qRT-PCR) analysis of gene expression levels of Cyclins and Cyclin-dependent kinases (CDKs) showed no significant difference among groups (Fig. S7a), and paralleled expression of osteogenic master genes was found, including *ALP* and *RUNX2*, as well as expression of stem cell activity-relevant markers, such as *Octamer-binding transcription factor 4* (*OCT4)*, *SRY-box transcription factor 2 (SOX2)*, and *NANOG* (Figs. S7a and S8a). Protein expression levels of the osteogenic markers, such as Collagen I (COL1), Osteopontin (OPN), RUNX2, and Osterix (OSX), were also comparable among unsorted, PDGFRA^+^, and PDGFRA^−^ DFSCs (Fig. S8b). Taken together, these findings suggest that PDGFRA^+^ DFSCs do not show in vitro functional superiority over PDGFRA^−^ DFSCs.

### Reciprocal interaction of PDGFRA^+^ dental MSCs with ECs potentially contributes to angiogenesis and osteogenesis

**Fig. 4:**
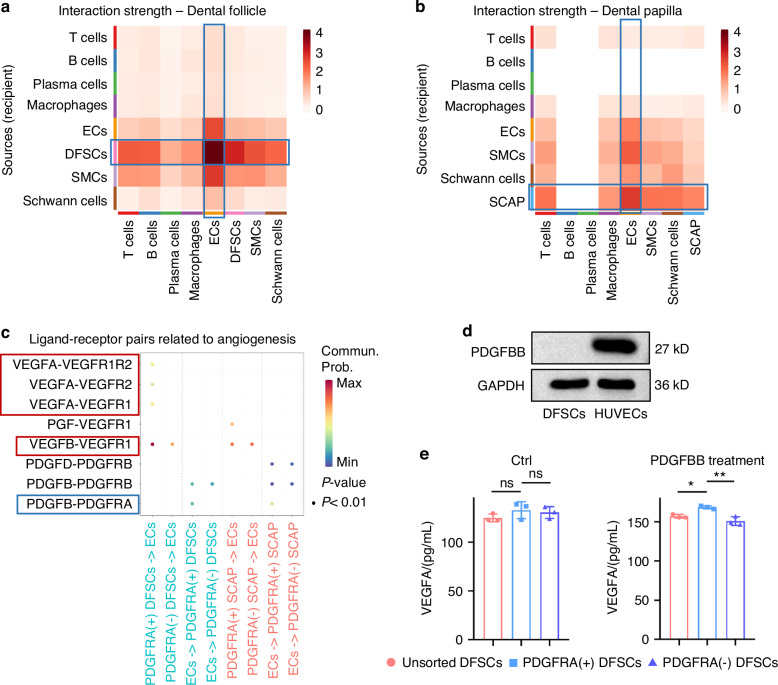
Reciprocal interaction of PDGFRA^+^ dental MSCs with ECs potentially contributes to angiogenesis and osteogenesis. **a** Interaction strength heatmap of dental follicle cell clusters. **b** Interaction strength heatmap of dental papilla cell clusters. **c** Dot plot showing significant ligand–receptor pairs related to angiogenesis between DFSCs and SCAP with ECs. **d** Western blot analysis of PDGFBB expression in DFSCs and HUVECs. **e** ELISA quantification of VEGFA concentrations in the conditioned medium from cultured DFSCs with and without PDGFBB treatment. Data were presented as mean ± SD. *n* = 3 per group. *P < 0.05; **P < 0.01; ns not significant (*P* > 0.05)

PDGFRA^+^ MSCs are known to reside in a perivascular region in a quiescent state,^31^ which receive P53-regulated signals from the arterial niche to maintain their lineage commitment in the mouse incisor.^32^ However, the paracrine mechanism governing MSC-vasculature crosstalk in tissue development remains elusive. Further investigation of the scRNA-seq data identified that the interaction strength between DFSCs and ECs was substantially higher than any other cell pairs within the tooth developmental microenvironment, which revealed predominant interactions between DFSCs and ECs in the dental follicle tissue (Fig. 4a). A similar pattern was observed for the interactions between SCAP and ECs, highlighting the significance for the further research (Fig. 4b). Upon particularly comparing the interaction pathways between PDGFRA^+^ DFSCs and PDGFRA^+^ SCAP with ECs, we discovered a consistent involvement of signaling related to angiogenesis and osteogenesis, including platelet-derived growth factor (PDGF) and vascular endothelial growth factor (VEGF) for angiogenesis, and bone morphogenetic protein (BMP) and WNT for osteogenesis (Fig. S9). The above information suggests crucial regulation of the coupling of angiogenesis-osteogenesis by the interaction of PDGFRA^+^ dental MSCs with ECs. Respective crosstalk of PDGFRA^+^ DFSCs and PDGFRA^+^ SCAP with ECs additionally indicated possible common and tissue-specific pathways, such as COLLAGEN for both MSC interaction with ECs, PERIOSTIN for PDGFRA^+^ DFSC-EC interaction, and Neural cell adhesion molecule (NCAM) for PDGFRA^+^ SCAP-EC communication (Fig. S9).

Moreover, ligand–receptor pair analysis predicted that the angiogenic contribution of PDGFRA^+^ DFSCs to ECs was mainly mediated through VEGF-VEGF receptor (VEGFR) pairs, particularly those involving the VEGFA (Fig. 4c). Reciprocally, PDGFRA^+^ DFSCs received PDGFBB signals from ECs, which was also indicated for PDGFRA^+^ SCAP (Fig. 4c). The osteogenic ligand–receptor pairs were additionally listed in detail (Fig. S10). The expression of PDGFBB by ECs, rather than dental MSCs, was validated at the protein level (Fig. 4d). To verify the reciprocal regulatory manner between PDGFRA^+^ DFSCs and ECs, we examined VEGFA secreted by DFSCs with and without PDGFBB treatment. We found that only after PDGFBB treatment, the concentration of VEGFA released by PDGFRA^+^ DFSCs was increased than unsorted and PDGFRA^−^ DFSCs (Fig. 4e). Mechanistically, the Kyoto encyclopedia of genes and genomes (KEGG) enrichment analysis comparing PDGFRA^+^ DFSCs and SCAP with their negative MSC counterparts indicated common and remarkable involvement of the phosphoinositide 3-kinase (PI3K)-protein kinase B (Akt) signaling downstream of PDGFRA activation (Fig. S11). Accordingly, Western blot analysis confirmed the upregulated phosphorylation of PI3K, Akt, and the downstream mammalian target of rapamycin (mTOR) after PDGFBB treatment in PDGFRA^+^ DFSCs over unsorted and PDGFRA^−^ DFSCs (Fig. S12). Taken together, these findings suggest that the reciprocal interaction of PDGFRA^+^ dental MSCs with ECs potentially contributes to angiogenesis and osteogenesis.

### Endothelial PDGFBB treatment improves the functionality of PDGFRA^+^ DFSCs

**Fig. 5:**
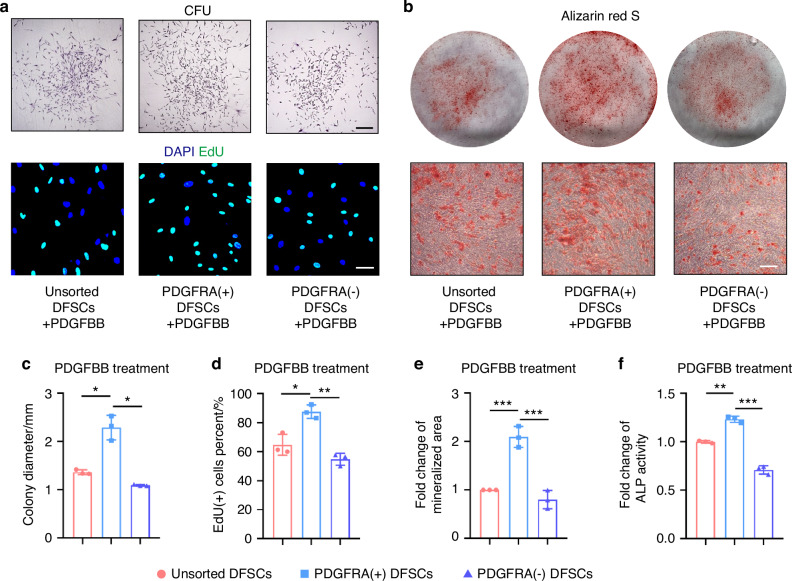
Endothelial PDGFBB treatment improves the functionality of PDGFRA^+^ DFSCs. **a** Representative images of colonies formed by DFSCs (scale bar = 250 μm) and EdU staining images showing DFSC proliferation (scale bar = 50 μm). **b** Alizarin red S staining images showing DFSC osteogenesis. Scale bar = 100 μm. **c** Quantification of colony diameter in CFU assay. **d** Quantification of EdU^+^ cells percent (%). **e** Quantification of fold change of mineralized area. **f** Quantification of the fold change of ALP activity. Data were presented as mean ± SD. *n* = 3 per group. *P < 0.05; **P < 0.01; ***P < 0.001; ns not significant (*P* > 0.05)

The above results prompted us to investigate whether PDGFBB treatment enhances the function of PDGFRA^+^ DFSCs. CFU analysis demonstrated that upon PDGFBB treatment, PDGFRA^+^ DFSCs exhibited superior self-renewal capacity over unsorted and PDGFRA^−^ DFSCs (Fig. 5a). Furthermore, PDGFBB treatment promoted the proliferation index of PDGFRA^+^ DFSCs, as shown by EdU staining (Fig. 5a). PDGFRA^+^ DFSCs also displayed enhanced mineralization potential after PDGFBB treatment (Fig. 5b). Statistical analysis confirmed the phenotypic findings, and examination of ALP activity additionally supported that PDGFBB safeguarded the osteogenic advantage of PDGFRA^+^ DFSCs over unsorted and PDGFRA^−^ DFSCs (Fig. 5c–f). Moreover, qRT-PCR analysis of cell cycle-related genes provided evidence of activated proliferation and stemness gene expression of PDGFRA^+^ DFSCs under PDGFBB (Fig. S7b), and enhanced osteogenic potential of PDGFRA^+^ DFSCs after PDGFBB treatment was verified at the mRNA and protein expression levels (Fig. S8c, d). Collectively, these results suggest that endothelial PDGFBB treatment improves the functionality of PDGFRA^+^ DFSCs.

### Paracrine promotion of CD31^+^EMCN^+^ vessel formation by PDGFRA^+^ DFSCs is safeguarded by endothelial PDGFBB

**Fig. 6:**
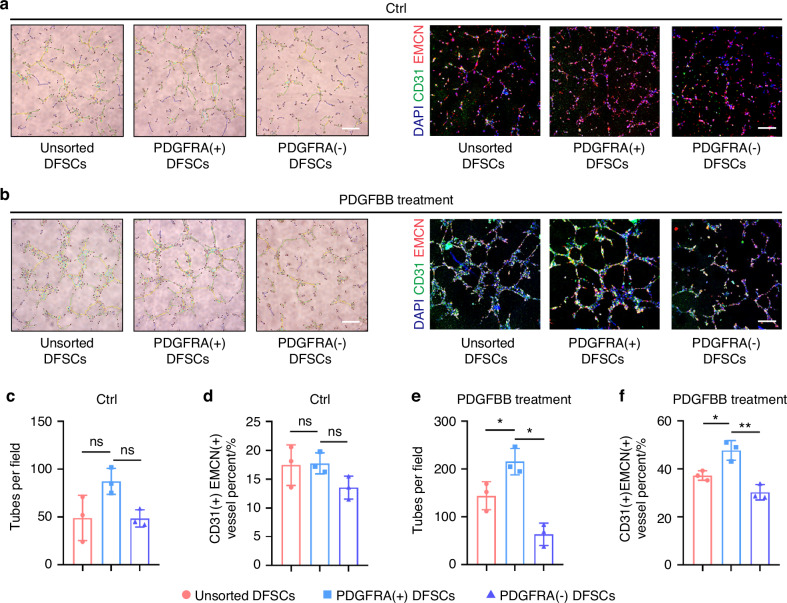
Paracrine promotion of CD31^+^EMCN^+^ vessel formation by PDGFRA^+^ DFSCs is safeguarded by endothelial PDGFBB. **a**, **b** Representative images showing tube formation of HUVECs treated by conditioned medium of DFSCs (scale bar = 250 μm) and IF staining images showing CD31^+^EMCN^+^ vessel formation of HUVECs treated by conditioned medium of DFSCs (scale bar = 200 μm). **c**, **e** Quantification of tube formation of HUVECs. **d**, **f** Quantification of CD31^+^EMCN^+^ vessel percent (%). DFSCs were pretreated with or without PDGFBB. Data were presented as mean ± SD. *n* = 3 per group. *P < 0.05; **P < 0.01; ns not significant (*P* > 0.05)

Next, we evaluated the reciprocal regulatory manner between PDGFRA^+^ DFSCs and ECs at the phenotypic functional level. To assess the paracrine potential of dental MSCs to facilitate EC angiogenesis, we collected conditioned medium of unsorted, PDGFRA^+^, and PDGFRA^−^ DFSCs with and without PDGFBB treatment, and applied the conditioned medium to cultured human umbilical vein endothelial cells (HUVECs), a known EPC line. In vitro scratch experiments demonstrated that DFSC effects on HUVEC migration were independent of PDGFRA expression per se, yet PDGFBB treatment promoted the capability of PDGFRA^+^ DFSCs to facilitate the migration of HUVECs in a paracrine mode (Fig. S13a–d). Tube formation assays further showed that PDGFBB treatment was indispensable for displaying the functional superiority of PDGFRA^+^ DFSCs over unsorted and PDGFRA^−^ DFSCs (Fig. 6a–d). Intriguingly, we noticed that the dental EC population in our scRNA-seq experiment was marked by concerted expression of CD31 and EMCN (Fig. S1a), and that CD31^+^EMCN^+^ vessels were especially considered to mediate the angiogenic-osteogenic coupling as a specialized capillary subtype, known as type H vessels in bone.^27,33^ Accordingly, we examined the capacity of DFSCs to regulate CD31^+^EMCN^+^ vessel formation. IF staining showed that the conditioned medium of PDGFRA^+^ DFSCs led to an increase in CD31^+^EMCN^+^ vessel percent only after pretreatment by PDGFBB (Fig. 6a, b, e, f). The Notch signaling pathway has been documented as the critical mechanism underlying type H vessel formation in bone.^34^ Expectedly, the active Notch intracellular domain, NICD, was only found upregulated by treatment with the conditioned medium from PDGFBB-stimulated PDGFRA^+^ DFSCs (Fig. S14a, b). Together, these findings suggest that paracrine promotion of CD31^+^EMCN^+^ vessel formation by PDGFRA^+^ DFSCs is governed by endothelial PDGFBB.

### PDGFRA^+^ DFSC aggregates promote the coupling of angiogenesis with osteogenesis and accelerate periodontal bone regeneration

**Fig. 7:**
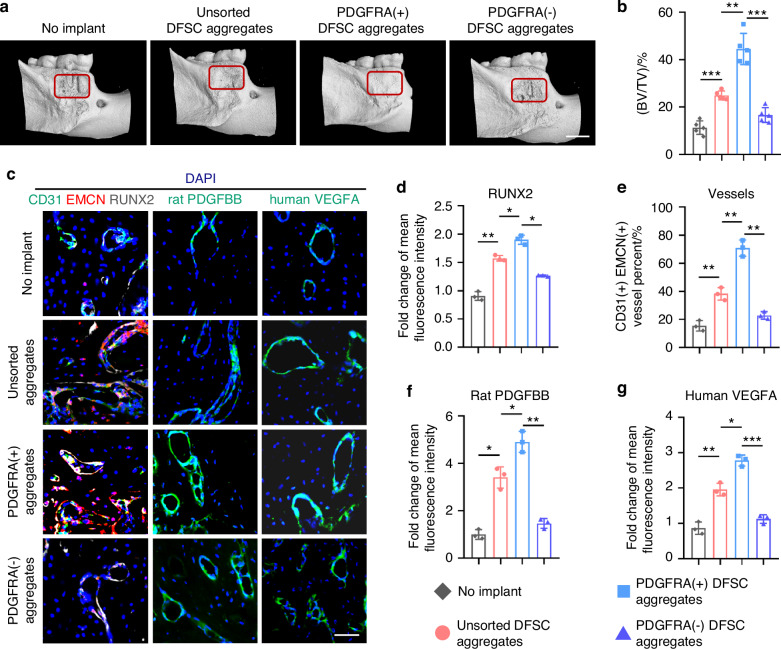
PDGFRA^+^ DFSC aggregates promote the coupling of angiogenesis with osteogenesis and accelerate periodontal bone regeneration. **a** Micro-CT images showing periodontal bone regeneration at 6 weeks after defect surgery and aggregate implantation in the original defect area indicated by red brackets. Scale bars = 2 mm. **b** Quantification of BV/TV in the original defect area. *n* = 5 per group. **c** IF staining images of RUNX2 (white) with CD31 (green) and EMCN (red), and rat PDGFBB (green) or human VEGFA (green), in the regenerated bone area. Scale bar = 50 μm. **d** Quantification of the fold change of mean fluorescence intensity of RUNX2. **e** Quantification of CD31^+^EMCN^+^ vessel percent (%). **f** Quantification of the fold change of mean fluorescence intensity of rat PDGFBB. **g** Quantification of the fold change of mean fluorescence intensity of human VEGFA. *n* = 3 per group. Data were presented as mean ± SD. *P < 0.05; **P < 0.01; ***P < 0.001

**Fig. 8:**
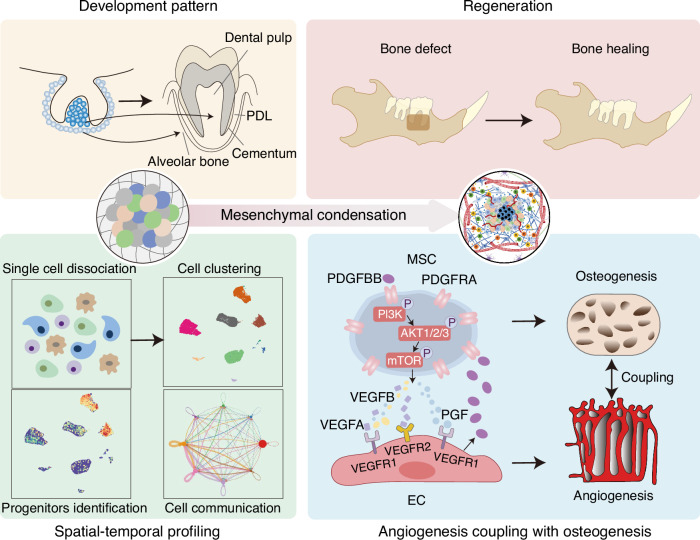
Graphical summary. The development pattern of mesenchymal condensation guides tissue regeneration that PDGFRA^+^ MSCs spatiotemporally communicate with ECs and safeguard angiogenesis-osteogenesis coupling

Finally, we investigated whether PDGFRA^+^ DFSCs harbor an optimized regenerative property and whether PDGFRA^+^ DFSCs essentially interact with ECs in regeneration. As a development-inspired approach, we established dental mesenchymal condensation-mimetic DFSC aggregates based on our previous protocol,^11^ which putatively shows odontogenic capacity with a favorable niche benefiting tissue regeneration.^8^ They were cultured with a high density and formed a membrane-like structure from a macroscopic view (Fig. S15a, b). H&E and Masson’s staining indicated that cell aggregates indeed exhibited continuous cell layers and rich ECM containing collagen deposited, especially in the PDGFRA^+^ group (Fig. S15c, d). Moreover, all aggregates exhibited only a slight amount of cell death after collection (Fig. S15e). It was further validated that the aggregation culture enabled a higher expression of *PDGFRA*, more significant expression of osteogenic genes, and higher release of VEGFA than the adherent culture (Fig. S16a, b). We respectively implanted aggregates formed by unsorted, PDGFRA^+^, and PDGFRA^−^ DFSCs into a periodontal bone defect model in rats, with non-implanted defects serving as the control (Fig. S17). At 6 weeks post-surgery, micro-computed tomography (micro-CT) scanning and 3D reconstruction uncovered that implantation of unsorted DFSC aggregates significantly promoted periodontal bone regeneration, whereas limited effects on periodontal bone repair were detected after implantation of PDGFRA^−^ DFSC aggregates (Figs. 7a, b and S18a). Importantly, periodontal bone defects implanted with PDGFRA^+^ DFSC aggregates displayed substantial regeneration, which almost restored the original bone mass in the defect region (Fig. 7a). Histological analysis by H&E and Masson’s staining confirmed the superior bone healing after implantation of PDGFRA^+^ DFSC aggregates, showing a remarkable replacement of defects with newly formed bone trabeculae, in contrast to the minimal bone formation and fibrous tissue presence in the PDGFRA^−^ DFSC aggregate group (Fig. S18b, c). IF staining further revealed that the osteogenic marker, RUNX2, was detected extremely adjacent to CD31^+^EMCN^+^ vasculature in the regenerated bone around bone canaliculi and lacunae area, suggesting coupling of angiogenesis with osteogenesis (Fig. 7c). Notably, significantly upregulated RUNX2 expression with an increased CD31^+^EMCN^+^ vessel percent was shown after implantation of PDGFRA^+^ DFSC aggregates, which demonstrated better recovery of the angiogenic-osteogenic coupling than unsorted and PDGFRA^−^ DFSC aggregates in periodontal bone regeneration (Fig. 7c–e). Moreover, IF staining of recipient rat PDGFBB and donor human VEGFA potentially indicated that effective donor-recipient interplay underlying PDGFRA^+^ DFSC aggregates-accelerated periodontal bone regeneration, in which implanted PDGFRA^+^ DFSC aggregates may respond to endothelial PDGFBB in the regenerative niche and reciprocally secrete VEGFA to promote angiogenesis, thus possibly forming a positive intercellular feedback loop to guarantee rapid regeneration (Fig. 7c, f, g). Collectively, these findings highlight that PDGFRA^+^ DFSC aggregates promote the coupling of angiogenesis with osteogenesis and accelerate periodontal bone regeneration. Taken all together, this study reveals a revolutionized development-inspired tissue regenerative strategy that spatiotemporal dissection of the odontogenic condensation at the single-cell level provides preferential stem cell populations with a communicative molecular basis for harnessing and safeguarding the angiogenic-osteogenic coupling-supported efficient bone defect healing (Fig. 8).

## Discussion

Regenerative medicine is now experiencing an evolving paradigm shift toward harnessing or recapitulating the developmental program for tissue and organ regeneration.^35^ Particularly, the organogenetic mesenchymal condensation has demonstrated remarkable promise to direct regenerative practice in animals and humans.^12,13,20,36^ However, how distinct cells are coordinated in condensation and regeneration remains unknown. In this study, we utilized scRNA-seq to delineate the profiles of postnatal developing dental tissues, revealing a common population of PDGFRA^+^ MSCs with significant odontogenic properties. Interestingly, through a series of bioinformatic and biological assays, we uncover that ECs serve as the critical component of the dental developmental niche, safeguarding the functionality of PDGFRA^+^ MSCs, and that PDGFRA^+^ MSCs are capable of inducing EPCs to form specialized CD31^+^EMCN^+^ vessels, which are mediated by reciprocal paracrine signals of VEGFA and PDGFBB. Our in vivo periodontal bone regeneration experiments have further provided compelling evidence that the crosstalk between PDGFRA^+^ DFSCs and ECs is pivotal for donor-recipient interplay to improve the efficacy of tissue regeneration. Collectively, these above findings underscore the significance of development-related interlineage progenitor cell communication in governing the odontogenic and regenerative potential, thereby paving the way for precise and efficient MSC-based regenerative therapeutics.

Tooth development is an intricate and prolonged biological process that encompasses migration, proliferation, condensation, differentiation, and intercellular communication of multilineage progenitor cells.^37–39^ During odontogenesis, a cohort of ectomesenchyme cells proliferate rapidly beneath the proliferative epithelium and form distinct condensations that include the dental follicle and the dental papilla.^8,40^ These developmental tissues persist until the tooth eruption in adulthood, yet their characteristics remain poorly understood.^17,41^ Particularly, despite previous reports on decoding dental tissues at the single-cell level, the composition and functionality of these human dental developing tissues have not been fully exploited.^37,42^ Furthermore, researchers have endeavored to dissect the developmental secrets of tooth formation for purifying candidate cells with superior regenerative capacity from heterogeneous cell clusters, having identified Msx1^+^ and SRY-box transcription factor 9 (Sox9)^+^ cells as the key ectomesenchymal dental niche cells driving tooth formation^38^ and CD24a^+^ and Placenta-specific 8 (Plac8)^+^ cells as multipotent stem cells for pulp development and regeneration.^17,24^ Our study builds upon these findings and further highlights PDGFRA^+^ MSCs as human dental progenitors, the regenerative potential of which is amplified through communication with EPCs/ECs, thus integrating the angiogenic and odontogenic/osteogenic processes. Our findings first provide a human dental tissue-derived specific MSC population for improved function, which holds great translational potential. While both DFSCs and SCAPs exhibit MSC properties, DFSCs may offer unique advantages for periodontal regeneration due to their developmental origin in the dental follicle as the precursors for periodontal tissues. Furthermore, although studies have reported that SCAP also mediates vascularized pulp tissue regeneration, it is still limited in confirming its application in hard tissue regeneration.^25,43^ With direct gene expression comparisons (VEGFA expression, etc.) being beyond our current data, future side-by-side comparisons of their angiogenic-osteogenic coupling efficiency would be valuable to investigate.

PDGFRA^+^ cells have been previously documented as progenitor cells derived from the mesoderm, somites, and the mesenchyme,^44^ with their role in embryonic development as regulators of the hematopoietic stem cell emergence from the hemogenic endothelium.^45^ PDGFRA^+^ perivascular cells have also been shown to differentiate into adipocytes,^46^ whereas MSX1^+^ mesenchymal progenitor cells with low expression of PDGFRA in the developing limb bud have the most potent capacity for cartilage regeneration.^47^ In tooth formation, Chai et al. have revealed PDGFA and PDGFRA as an autocrine mechanism to mediate continued epithelial-mesenchymal interaction during odontogenesis, which is indispensable for tooth size and cusp morphogenesis.^48,49^ Recently, they have further documented that PDGFRA^+^ and PDGFRB^+^ cells differentially contribute to defined cell lineages in the adult mouse incisor by accepting PDGF ligands from arteries deposited in the MSC region.^32^ Therefore, with tissue-specific differences in PDGFRA^+^ cell characteristics, microenvironmental effects regulating these mesenchymal progenitors and the implications require elucidation. In the present study, we discovered that PDGFRA hallmarks the most promising stem cell population within the general dental progenitor cells, providing advantages for regeneration applications that are attributable to their strong specificity and high proportion. Further, we show that PDGFRA^+^ DFSCs interact closely with ECs via exchanging VEGFA and PDGFBB, which safeguards tissue regeneration as a crucial mechanism. Future works are still needed to directly clarify in vivo the donor-recipient interplay with advanced tracing and imaging techniques.

The establishment and evolution of mesenchymal condensation is a complex process and involves the coordination of multilineage progenitors.^50,51^ MSCs, with their mechanical property of aggregation and multipotent differentiation potential, play key roles in influencing various progenitor cells to collaborate in forming organogenetic condensations and facilitating subsequent development.^20^ Here, we further reveal that the potency of PDGFRA^+^ DFSCs is enhanced by paracrine signals from ECs, which release PDGFBB to activate PDGFRA. In accordance, MSC-driven co-aggregation strategies involving EPCs have been established to build immature tissue constructs for improved vascularization during efficient regenerative performance.^20,52^ Beyond intercellular communication, mesenchymal-endothelial transition has been reported in the bioengineering context, with MSCs capable of being mechanically and pharmacologically conditioned to differentiate into ECs or pericytes, thus participating in the stabilization and maturation of vessels.^53^ Interestingly, a novel mechanism regulating the endothelial-mesenchymal transition has been identified, offering insights into the crosstalk between ECs and MSCs and their implications for heart valve development.^54^ However, the direct endothelial-mesenchymal transition has been denied in hard tissue development in the bone marrow, adding complexity to this field.^55^ Besides, cell-type annotation of the dental follicle tissue indicates a significant presence of immune cells. Although previous scRNA-seq studies have illuminated the immune microenvironment in diseased status and the interaction patterns of immune cells,^37,56^ the role of MSC interaction with immune cells during tissue development and their potential value in regenerative therapies still need deeper exploration.

Periodontal bone tissues, in conjunction with the periodontal ligament, serve as a critical anchorage that firmly connects teeth to the jaw bone, thereby facilitating their function and maintaining oral health.^8,57^ In the clinic, patients frequently encounter periodontal bone defects that lead to tooth loss in severe cases, which significantly impair the functionality of the stomatognathic system, affect facial esthetics, and even result in gastrointestinal issues and psychological disorders.^57,58^ Therefore, it is imperative to explore effective solutions for periodontal bone regeneration. However, repair of the lost periodontal tissue remains unstable by current regenerative therapies, including barrier membranes, grafting materials, growth factors, or their combinations.^59,60^ Intriguingly, the periodontium originates from the dental follicle condensation in odontogenesis,^8^ and encouraged by this natural process, DFSC aggregates have been widely employed in dental tissue regeneration in animal studies.^41,61,62^ Nevertheless, our previous clinical study discovered that implantation of MSC aggregates in the diseased recipient status fails to rescue periodontal bone loss despite safety, which requires an optimized strategy to promote regenerative efficacy.^18^ In this study, our in vivo experiments confirm that implanted PDGFRA^+^ cell aggregates persist in the recipient microenvironment, secrete factors aiding in angiogenesis, and potentially stimulate ECs to release PDGFBB for their own functional maintenance. This reciprocal communication activates a cascade that synergistically enhances angiogenesis and osteogenesis in rapidly repairing the periodontal defect. It cannot be neglected that in the recipient periodontal microenvironment without implanted aggregates, ECs exhibited significantly lower PDGFBB secretion and poorer angiogenic effects. Additionally, researchers have proposed that implanted cell aggregates may release extracellular vesicles to modulate recipient stem cell proliferation and angiogenesis,^28,63,64^ while apoptosis of implanted cell aggregates might also be inevitably needed for regeneration.^65^ Further investigation into the fates and mechanisms of PDGFRA^+^ cell aggregates promoting regeneration in the recipient microenvironment is necessary in the next step.

Importantly, it cannot be overlooked that HUVECs and local vascular ECs in the periodontal bone tissue may exhibit certain differences in biological characteristics. Although our study suggests that ECs play a pivotal role in vascularization, bone regeneration involves a more complex and finely regulated coupling of angiogenesis and osteogenesis.^66–68^ Whether and how HUVECs can completely and accurately replicate this process still requires further evidence in future studies. In addition, although the periodontal bone was successfully regenerated by sorted PDGFRA^+^ DFSC aggregates efficiently in the study, the application of PDGFRA^+^ DFSC aggregates in clinical regeneration will still encounter many difficulties. For example, obtaining sufficient sources of the PDGFRA^+^ cell subpopulation to achieve large-scale clinical application remains a key issue. In recent years, the 3D culture system has been developed to facilitate the long-term maintenance of progenitor cell stemness and enhance the regenerative capacity of stem cells in tissue repair.^69,70^ Based on 3D cultures, organoid technology has profoundly impacted various fields, especially in regenerative medicine.^71^ Significantly, the establishment of stem cell banks has emerged as a promising approach, enabling the pre-storage and sharing of stem cell resources and offering a dependable solution to address critical demands. Further studies are needed to investigate how to ensure a sufficient cell source to establish a standard system in the clinical application of PDGFRA^+^ DFSC aggregates.

In summary, our study unravels a specialized developmental mesenchymal-endothelial interplay related to odontogenic condensation that inspires and contributes to efficient tissue regeneration. These findings will benefit feasible translational strategies for clinical tissue repair.

## Materials and methods

### Human tissue harvest and preparation

All donors were patients in the School of Stomatology, The Fourth Military Medical University, and have signed informed consent to this study. Experimental procedures of human samples were approved by the Ethics Committee of The Fourth Military Medical University with the approval number IRB-REV-2022187. Dental follicles were harvested based on inclusion criteria as follows: patients were aged 20 ± 2 years old, had no history of pain or infection, and had unerupted third molars with healthy surrounding tissues. Dental papilla tissues were acquired from the third molars that had not completely developed roots with open apical foramen. All the patients were examined by oral panorama before surgery, and any of the third molars with an abnormal low-density shadow around were excluded. After local anesthesia, incising and flap elevation were performed to expose the bone covering the teeth. Fissure burs assembled in a high-speed drill were used to remove a portion of bone and provide space for elevator usage. After tooth extraction, the wound was closed carefully, and patients were given instructions for post-operative care. Dental tissues attached to the teeth were immersed in 10% alpha-minimum essential medium (α-MEM; 12571-048, Invitrogen, USA) and immediately delivered to the laboratory in an ice box. Dental follicles were obtained by cutting off soft tissues around teeth, and dental papilla were isolated by amputation of soft tissues out of the root apex. The tissues were sectioned into 2 mm³ pieces and rinsed twice with phosphate-buffered saline (PBS; P5493, Sigma-Aldrich, USA) before undergoing the following experiments.

### ScRNA-seq analysis

Dental tissues were initially dissociated into single cells using 0.02% Type I collagenase (17018029, Gibco, USA). Cells were barcoded with 10× gel beads and encapsulated in oil to form single-cell gel beads-in-emulsion (GEMs). Reverse transcription reactions were engaged in barcoded full-length cDNA, followed by the disruption of emulsions using the recovery agent. Single-cell 3′ Reagent v3 Kits (1000268, 10× Genomics, USA) were used for scRNA-seq library construction. The sequencing was performed on the Illumina Nova 6000 PE150 platform (Illumina, USA). Cell Ranger (version 7.0.1) software (10× Genomics, USA) was employed for quality control and through comparisons between reads to the genome by the spliced transcript alignment to a reference aligner. The cells that met the criteria were retained: (1) gene numbers > 200, unique multiplex index (UMI) > 1 000, and log_10_GenesPerUMI > 0.7; (2) the UMI of mitochondrial genes < 15% and hemoglobin genes < 5%. To mitigate batch effects, mutual nearest neighbor (MNN) analysis was conducted. Cells were then clustered using the MNN clustering algorithm and visualized using the two-dimensional uniform manifold approximation and projection (UMAP) algorithm. For a detailed identification of cell types, homotypic clusters were selected for re-analysis, graph-based clustering, and marker analysis. The top 100 genes of DFSCs and SCAP, ranked according to gene differences, were analyzed and displayed as a Venn plot performed on the OECloud tools at https://cloud.oebiotech.com. The dot plots, feature plots, and violin plots of genes were also performed on the OECloud tools at https://cloud.oebiotech.com. The violin plots of gene expression level were drawn using ggplot2 in the R package (4.0.3). GO and KEGG enrichment analysis was performed using ClusterProfiler of the R package, and an adjusted *P* value < 0.05 was set as a limitation (ListHitså 2). RNA velocity analysis was performed using the Python script velocyto.py on the Cell Ranger output folder to elucidate the dynamics of cell subpopulations. The results were projected to UMAP and visualized using Seurat software. PDGFRA^+^ DFSCs were counted by threshold > 0. Cell-cell communication was conducted using CellChat (version 1.1.3) to examine interactions involving ligands, receptors, and their cofactors. After standardizing of expression matrix and creating cellchat objects, preprocessing was performed using default parameters. Potential ligand–receptor interactions were calculated, and the aggregateNet function was utilized to achieve the intercellular communication networks aggregated.

### Cell cultures

Dental tissues were obtained as mentioned above, cut into pieces, and digested with 0.02% Type I Collagenase for 1 h (17018029, Gibco, USA). Primary DFSCs and SCAP were cultivated in α-MEM supplemented with 10% fetal bovine serum (FBS; Gibco, USA), 2 mmol/L L-glutamine (35050061, Invitrogen, USA), 100 μg/mL penicillin, and 100 IU/mL streptomycin (15070063, both from Invitrogen, USA).

HUVECs were sourced from iCell Bioscience Inc. (Shanghai, China) and maintained in an EC medium (1001, ScienCell, USA). Cells were passaged using a 0.25% trypsin solution (15050057, Gibco, USA) as they reached 80%–90% confluence. Culture conditions were maintained at 37 °C in a 5% CO_2_ atmosphere, with medium changes every 3 days.

### Flow cytometric analysis

Cultured DFSCs and SCAP were harvested and stained with an anti-human CD90-PE antibody (5 µL per test, 12-0909-41, eBioscience, USA), CD90-FITC antibody (5 µL per test, 11-0909-42, eBioscience, USA), and an anti-human CD45-PE antibody (5 µL per test, 304058, Biolegend, USA), respectively. Samples were incubated at 4 °C in the dark for 1 h, filtered, and prepared for analysis using a flow cytometer (CytoFLEX, Beckman, USA). To validate the proportion of PDGFRA^+^ cells in dental follicle tissue cells and cultured DFSCs, cells were stained with an anti-human PDGFRA-PE antibody (5 µL per test, sc-398206 PE, Santa Cruz Biotechnology, USA) and were analyzed using a flow cytometer (CytoFLEX, Beckman, USA) after 1-h incubation at 4 °C in the dark.

### CFU assay

DFSCs and SCAP were plated and cultured in 6-well dishes (Corning, USA) at a density of 1000 cells per well. After 14 days, cells were fixed with 4% paraformaldehyde (PFA; 1004965000, Sigma-Aldrich, USA) and stained with 1% crystal violet (C8470, Solarbio, China) for 5 min. The experiment was performed thrice to ensure the reliability and consistency of results.

### EdU assay

DFSCs and SCAP were seeded in 24-well plates (Corning, USA) at 1 × 10^4^ cells per well and incubated for 24 h. Cells were then treated with EdU at a working concentration of 10 μmol/L in 200 μL of culture medium for 48 h. Afterward, the cells were detected using a VF 488 Click-iT EdU universal cell proliferation detection kit (HY-K1087, MCE, China) according to the manufacturer’s instructions.

### Osteogenic and adipogenic differentiation

DFSCs and SCAP were seeded in 6-well plates at a density of 3 × 10^5^ each per well. The culture medium was replaced with the osteogenic medium or the adipogenic medium until full confluence was achieved. The osteogenic medium was prepared, consisting of α-MEM supplemented with 10% FBS, 5 mmol/L β-glycerophosphate (G5422, Sigma-Aldrich, USA), 10 nmol/L dexamethasone (D4902, Sigma-Aldrich, USA), and 50 μg/mL ascorbic acid (02100769-CF, MP Biomedicals, USA). The adipogenic medium was prepared consisting of α-MEM supplemented with 10% FBS, 200 μmol/L indomethacin (I8280, Sigma-Aldrich, USA), 2 mmol/L insulin (I3536, Sigma-Aldrich, USA), 0.5 mmol/L isobutylmethylxanthine (IBMX; I5879, Sigma-Aldrich, USA), and 10 nmol/L dexamethasone (D4902, Sigma-Aldrich, USA). The medium was refreshed every 3 days. After induction for 14 days, cells were fixed with 4% PFA for 30 min. Mineralized nodules were stained with a 1% alizarin red S solution (60504ES25, Yeasen, China), and lipid droplets were stained with a 0.3% oil red O solution (O104972, Aladdin, China) at room temperature for 20 min. After rinsing with PBS twice, photographs were captured under a bright field of fluorescence microscope (Olympus, Japan) or using an inverted microscope (Leica, Germany). For the detection of ALP activity, supernatants from cell cultures of each group were collected, and ALP activity was detected using an ALP activity assay kit (A059-2, Nanjingjiancheng, China) according to the manufacturer’s instructions. The absorbance of each well was determined by measurements at 520 nm.

### Magnetic cell sorting

DFSCs of the first passage were used for sorting. Single-cell suspension was prepared and incubated with 2 μL of a biotinylated CD140a (i.e., PDGFRA) monoclonal antibody (13-1401-80, Themofisher, USA) at room temperature for 10 min. Cells were then conjugated with MagniSort™ Streptavidin Positive Selection Beads (MSPB-6003-74, Themofisher, USA) according to the manufacturer’s instructions. After being washed with the separation buffer, cells were subjected to a magnetic field to separate positive and negative populations.

### ELISA

The conditioned medium was collected and centrifuged at 4 °C for 20 min at a speed of 3 000 r/min. The VEGFA concentrations were measured using a commercial ELISA kit (F111309-A, FANKEW, China) according to the manufacturer’s instructions.

### qRT-PCR

Total RNA was extracted using the MIsZOL Reagent (MI00617, Mishushengwu, China). 5× PrimeScript RT Master Mix (RR036A-1, Takara, Japan) was used for cDNA synthesis, and reverse transcription proceeded on the PCR Amplifier (EasyCycler, Germany). qPCR was performed using TB Green® Premix Ex Taq (RR420A, Takara, Japan), and gene expression was detected using a Real-Time System (Bio-Rad, USA). The CFX Manager software (Bio-Rad, USA) was utilized for result analysis. The primer sequences (5′–3′) were listed as: *h-PDGFRA* forward: AAGAGATCATTGGAGGCCGTG; reverse: AGGATTAGGCTCAGCCCTGT; *h-CCND2* forward: *GGTCATCCTTGGTCTATGTGCTCTG;* reverse: GGGTTGTCTTCTCCTCTGGCTTTG; *h-CCND3* forward: ACGAGGAGGTATGTGAGGAGCAG; reverse: AGACAGGTAGCGATCCAGGTAGTTC; *h-CDK2* forward: AGGATGTGACCAAGCCAGTACCC; reverse: CCACCTGAGTCCAAATAGCCCAAG; *h-CDK3* forward: AGGAGGGAGTGAGGGAGAGGAG; reverse: AGCACATCTCAGGTGAAGGAACAAC; *h-ALP* forward: TAAGGACATCGCCTACCAGCTC; reverse: TCTTCCAGGTGTCAACGAGGT; h-RUNX2 forward: CTTTACTTACACCCCGCCAGTC; reverse: AGAGATATGGAGTGCTGGTC; *h-OCT4* forward: GCCGTATGAGTTCTGTGGGG; reverse: CTCCTTCTCCAGCTTCACGG; h-SOX2 forward: ACACCAATCCCATCCACACT; reverse: GCAAACTTCCTGCAAAGCTC; *h-NANOG* forward: TGAACCTCAGCTACAAACAG; reverse: CTGGATGTTCTGGGTCTGGT; *h-GAPDH* forward: CTTTGGTATCGTGGAAGGACTC; reverse: GTAGAGGCAGGGATGATGTTCT.

### Cell migration assay

HUVECs were seeded into 6-well plates at a density of 5 × 10^5^ cells per well. At 90% confluence, scratches were created using sterile pipette tips. Conditioned medium from DFSCs was collected, filtered through 0.22 µm filters, and mixed with fresh and serum-free EC medium at a 1:1 ratio before addition. Scratch areas were photographed at 0, 12, and 24 h using an inverted microscope (Leica, Germany) and quantified using the ImageJ software (NIH, USA).

### Tube formation assay

Capillary-like network formation was assessed through an in vitro tube formation assay on μ-Slides 15 Well 3D (81506, ibidi, Germany), precoated with Matrigel (082703, Shanghai Nova Medical Technology, China). HUVECs were seeded at 1 × 10^4^ cells per well. Conditioned medium from DFSCs was collected, filtered through 0.22 µm filters, and mixed with fresh and serum-free EC medium at a 1:1 ratio before addition. Tube formation was observed and photographed after 5 h using an inverted microscope (Leica, Germany), and the number of network structures was quantified using the ImageJ software (NIH, USA).

### PDGFBB treatment

All experiments involving DFSCs treated with PDGFBB were conducted by supplementing the culture medium with PDGFBB (HY-P7055, MCE, China) at a concentration of 100 ng/mL when the cell confluence reached 40%-50%. After 48 h, conditioned medium was collected for indirect coculture with ECs and deposited at −80 °C. Additionally, the DFSCs were applied for qRT-PCR and western blot analysis. As for osteogenic differentiation assays, the medium was refreshed along with PDGFBB at the same concentration of 100 ng/mL.

### Aggregate culture

For the induction of cell aggregates, DFSCs were seeded into 24-well plates at a density of 1 × 10^4^ cells per well. Once cells reached 80% confluence, the medium was replaced with α-MEM supplemented with 50 μg/mL ascorbic acid (02100769-CF, MP Biomedicals, USA), 2 mmol/L L-glutamine (35050061, Invitrogen, USA), 100 μg/mL penicillin, and 100 IU/mL streptomycin (15070063, both from Invitrogen, USA). The medium was refreshed every 2 days. After ~7 days, cell aggregates with white membrane-like structures were formed and were acquired from the culture plates using a cell scraper.

### Live/Dead cell staining

Aggregates induced in the 6-well plate were washed with PBS twice. According to the kit instructions (C2015M, Beyotime, China), the staining working solution was added and incubated at 37 °C in the dark for 30 min. Subsequently, counterstain the nuclei with DAPI. After washing with PBS, observe and capture images under a fluorescence microscope.

### Rats

Fifty male Sprague-Dawley rats, aged 8 weeks with weighing 200–220 g, were provided by the Laboratory Animal Centers of The Fourth Military Medical University. The animals were housed in a well-ventilated environment with a standard 12-h light-dark cycle and free access to water and food. All animal experiments were performed in compliance with relevant laws and ethical regulations, following ARRIVE guidelines, and approved by the Ethics Committee of The Fourth Military Medical University with the approval number IRB-REV-2022187.

Rats were anesthetized using 3% isoflurane via an anesthesia machine (RWD, China), and the right mandibles were exposed. A box-type periodontal window defect (3 × 2 × 1 mm^3^ in width, height, and depth, respectively) at the buccal side of the first molar was surgically created using a 0.8 mm diameter drill. Following the establishment of defects, DFSC aggregates were implanted, and defects without any implantation served as blank controls. At 6 weeks post-surgery, the animals were euthanized using CO_2_, and their mandibles were harvested for further analysis.

### Micro-CT analysis

For quantitative assessment of periodontal bone regeneration, the right mandibles were meticulously excised and fixed in 4% PFA after the removal of soft tissues. The specimens were then prepared for micro-CT scanning using a PerkinElmer^TM^ Quantum GX2 micro-CT device (PerkinElmer, USA) at the Animal Center of The Fourth Military Medical University. Scanning parameters included an energy setting of 90 kV and a current of 80 μA, with a resolution of 18 μm. The acquired data were processed for 3D reconstruction using the VG Studio MAX software (version 2023.2.1) (Volume Graphics, Germany). Bone volume over tissue volume (BV/TV) was calculated to provide a quantitative analysis of the regenerated bone.

### Histological analysis

Dental follicles, dental papilla, and cell aggregates were fixed in 4% PFA for 12 h. Rat mandibles were harvested and fixed in a 4% PFA for 24 h, followed by decalcification in 17% ethylenediaminetetraacetic acid (EDTA) for 4 weeks. The dental follicle, dental papilla, cell aggregates, and decalcified samples underwent a series of ethanol and xylene for dehydration before being embedded in paraffin. Sections of 5 μm thickness were meticulously prepared using a microtome (Leica, USA). H&E staining and Masson’s staining were performed according to established protocols.^64,72^ Images were taken by the SLIDEVIEW VS200 (Olympus, Japan).

### IF staining

For cultured DFSC and SCAP, cells were washed twice with PBS and fixed with 4% PFA at room temperature for 30 min. Fixed cells were permeabilized with 0.5% Triton X-100 (X100PC, Sigma-Aldrich, USA) for 15 min. Cells were then blocked in 5% bovine serum albumin (BSA; 0881066-CF, MP Biomedicals, USA) in PBS for 30 min at room temperature. Cells were stained with primary antibodies for Nestin (sc-33677, Santa Cruz Biotechnology, USA; diluted 1:100), CK14 (ab7800, Abcam, UK; diluted (1:100), and PDGFRA (sc-398206 PE, Santa Cruz Biotechnology, USA; diluted 1:100), overnight at 4 °C, and then washed three times with PBS. Secondary antibodies were stained for 1 h at room temperature and then washed three times with PBS before DAPI staining (ab104139, Abcam, UK) for 5 min. Cells were examined under a fluorescence microscope (Olympus, Japan).

For HUVECs, after tube formation on the µ-Slides, capillary-like structures were fixed with 4% PFA for 30 min, followed by rinsing with PBS and blocking in 5% BSA for 30 min at room temperature. Then, HUVECs were incubated with primary antibodies for CD31 (FAB3628G, R&D Systems, USA; diluted 1:100) and EMCN (DF13357, Affinity, China; diluted 1:100) at 4 °C overnight. Fluorescence-conjugated secondary antibodies were then stained at 37 °C for 1 h, followed by DAPI staining for 10 min. The fluorescent images were captured with a confocal laser scanning microscope (CLSM) (Nikon, Japan). The percentage of CD31^+^EMCN^+^ vessels was analyzed by the ImageJ software (NIH, USA).

For PFA-fixed human dental follicles, dental papilla, and decalcified mouse mandibles, tissues were dehydrated in 30% sucrose solution (Sigma-Aldrich, USA) and embedded in the O.C.T compound (4583, Sakura Finetek, USA) to obtain 10 μm frozen sections in a freezing microtome (Leica, Germany). After permeabilization with 0.3% Triton X-100 (X100PC, Sigma-Aldrich, USA) for 15 min and blocking with the goat serum (AR0009, BOSTER, China) for 30 min at room temperature, sections were incubated with primary antibodies for PDGFRA (sc-398206 PE, Santa Cruz Biotechnology, USA; diluted 1:100), MSX1 (bs-8512R, Bioss, China; diluted 1:100), PAX9 (A19741, ABclonal, China; diluted 1:100), RUNX2 (sc-390715, Santa Cruz biotechnology, USA; diluted 1:100), CD31 (FAB3628G, R&D Systems, USA; diluted 1:100), EMCN (DF13357, Affinity, China; diluted 1:100), rat PDGFBB (ab21234, Abcam, UK; 1:100), and human VEGFA (ab9570, Abcam, UK; 1:100) at 4 °C overnight. Fluorescence-conjugated secondary antibodies were then stained at 37 °C for 1 h, followed by DAPI staining for 10 min. The fluorescent images were captured with CLSM (Nikon, Japan).

### Western blot analysis

Proteins were extracted on ice using a RIPA buffer (P0013C, Beyotime, China) supplemented with protease inhibitors for 10 min. After protein quantification using a BCA assay (PA115-02, TIANGEN, China), 20 μg proteins were loaded onto sodium dodecyl sulfate-polyacrylamide gels and underwent electrophoresis, followed by transferred onto polyvinylidene fluoride membranes (GVWP02500, Roche, Switzerland) and blocked in 5% BSA (0881066-CF, MP Biomedicals, USA) for 1.5 h at room temperature. The membranes were incubated overnight at 4 °C with primary antibodies diluted in PBS containing 0.1% Tween 20 (PBST). Then membranes were washed 3 times with PBST and incubated at room temperature with secondary antibodies for 1.5 h. After further washing with PBST, the protein bands were visualized with an enhanced chemiluminescence kit (4AW011-100, 4A Biotech, China) and evaluated with a gel imaging system (4600, Tanon, China). The primary antibodies used were: anti-COLI antibody (ab260043, Abcam, UK; diluted (1:1 000), anti-OSX antibody (ab209484, Abcam, UK; diluted 1:1 000), anti-OPN antibody (22952-1-AP, Proteintech, China; diluted 1:1 000), anti-RUNX2 antibody (12556, Cell Signaling Technology, USA; diluted 1:1 000), anti-PDGFBB antibody (ab16829, Abcam, UK; diluted 1:1 000), anti-p-PI3K antibody (AF3242, Affinity, China; diluted 1:1 000), anti-PI3K antibody (AF5112, Affinity, China; diluted 1:1 000), anti-p-AKT antibody (AF0016, Affinity, China; diluted 1:1 000), anti-AKT antibody (ab179463, Abcam, UK; diluted 1:1 000), anti-p-mTOR antibody (5536T, Cell Signaling Technology, USA; diluted 1:1 000), anti-mTOR antibody (2983T, Cell Signaling Technology, USA; diluted 1:1 000), anti-NICD antibody (3608S, Cell Signaling Technology, USA; diluted 1:1 000), and anti-GAPDH antibody (30201ES20, Yeasen, China; diluted 1:2 000). The secondary antibodies included Peroxidase-AffiniPure Goat Anti-Mouse IgG (H+L) (115-035-003, Jackson ImmunoResearch, USA) and Peroxidase-AffiniPure Goat Anti-Rabbit IgG (H+L) (111-035-003, Jackson ImmunoResearch, USA), and the secondary antibody dilute ratio was 1:10 000.

### Statistical analysis

All data were presented as mean ± standard deviation (SD). The normality and variances of all data were evaluated before comparisons, and all data matched the normal distribution. As for two samples, comparisons were made using unpaired two-tailed Student’s *t*-tests for data with equal variances, and Welch’s correction was employed when equal variances were not assumed. Multiple samples were compared using ordinary one-way analysis of variance (ANOVA) with Tukey’s post-hoc tests when equal variances were assumed. Brown-Forsythe and Welch ANOVA tests with Games-Howell’s multiple comparisons tests were employed when equal variances were not assumed. For all experiments, *P* < 0.05 was considered to be significant. The GraphPad Prism software (Version 8.0.1) was used for all statistical analysis.

## Supplementary information

Supplementary Figures and Legends

Supplementary Information

Supplementary Table S1

## Data Availability

The raw sequence data reported in this research have been deposited in the Genome Sequence Archive^73^ in the National Genomics Data Center,^74^ China National Center for Bioinformation/Beijing Institute of Genomics, Chinese Academy of Sciences (GSA-Human: HRA008022) that will be publicly accessible upon publication at https://ngdc.cncb.ac.cn/gsa-human. Any additional information required to reanalyze the data reported in this work is available from the lead contact upon reasonable request.
