# Supplementary material for: Single-cell transcriptomics identifies PDGFRA+ progenitors orchestrating angiogenesis and periodontal tissue regeneration
Source: Int J Oral Sci. 2025 Jul 24;17:56. doi: 10.1038/s41368-025-00384-6 (PMC12289874; doi:10.1038/s41368-025-00384-6)
Supplement: Supplementary file 1 — Supplementary Figures and Legends [file 41368_2025_384_MOESM1_ESM.docx]

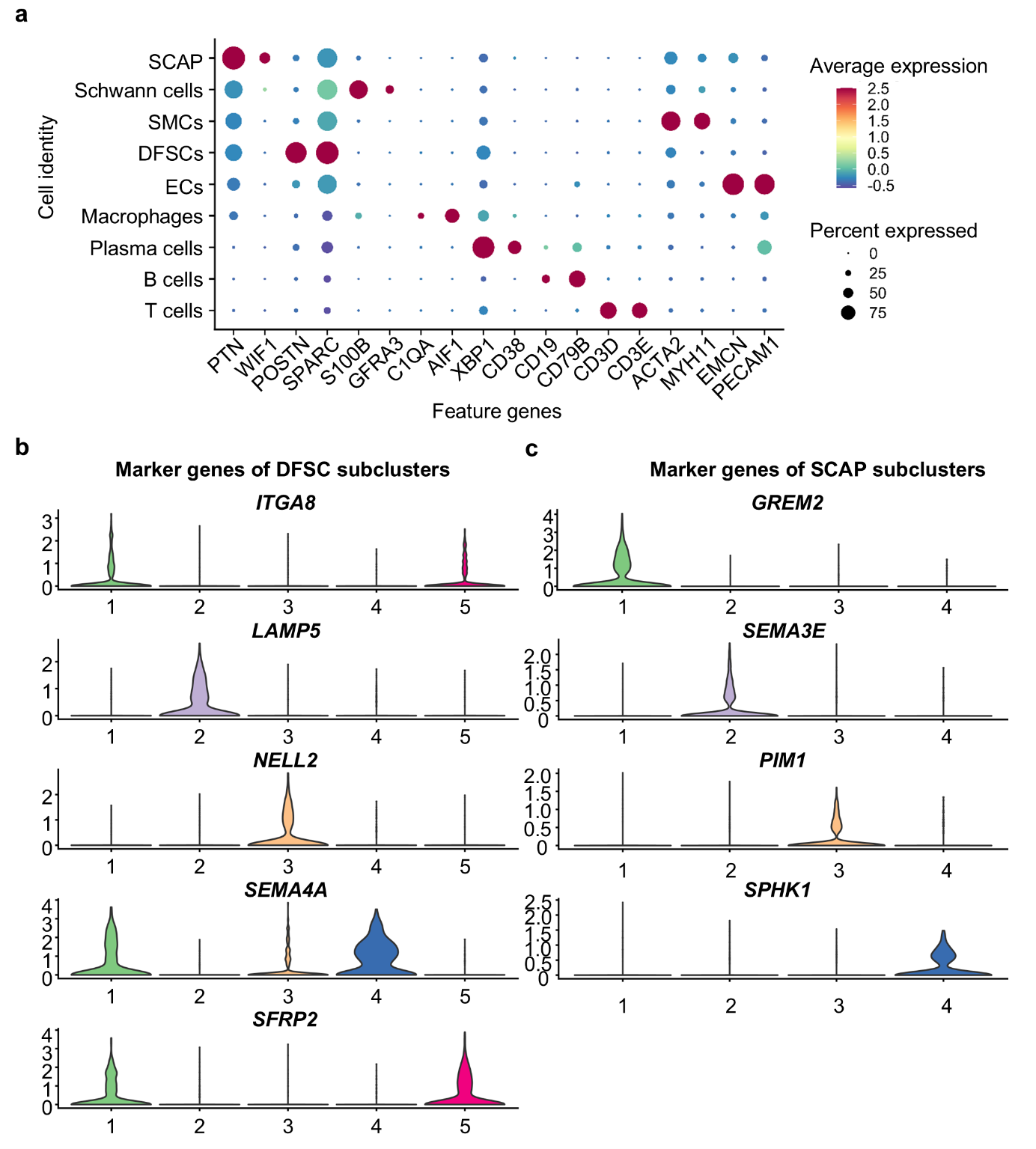


**Figure S1. Representative gene markers for cell clusters.** (a) Feature plot of unbiasedly identified cell clusters based on the expression of selected marker genes. The color intensity reflects the average gene expression level in each cluster. (b and c) Violin plots of distinct expression of selected marker genes in each subcluster of DFSCs and SCAP.

**
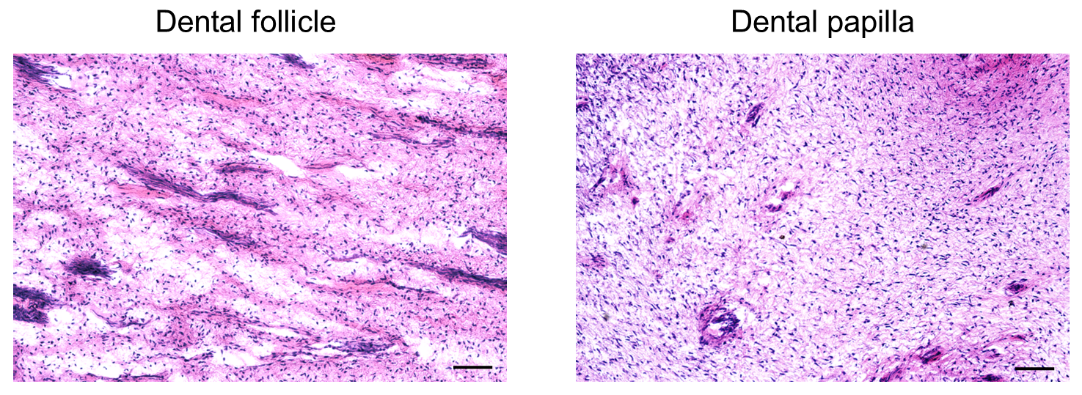
**

**Figure S2. H&E staining images of human dental follicle and dental papilla.** Dental follicle (left) and dental papilla (right). Scale bar = 100 μm.


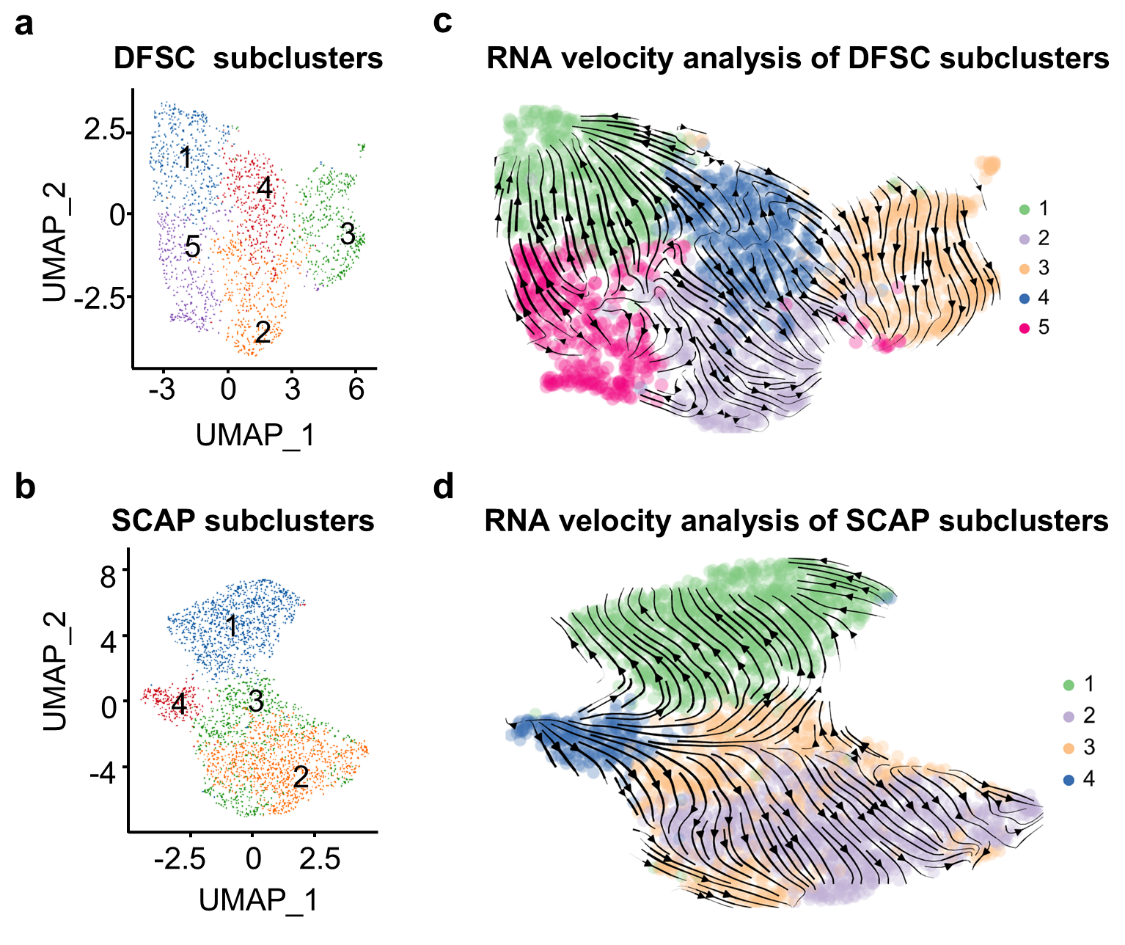


**Figure S3.** **Characteristics of DFSC and SCAP subclusters.** (a) UMAP plot of 1,858 DFSCs showing subclusters. (b) UMAP plot of 2,840 SCAP showing subclusters. (c) RNA velocity analysis for the pseudotime trajectories of DFSC subclusters. (d) RNA velocity analysis for the pseudotime trajectories of SCAP subclusters.

**
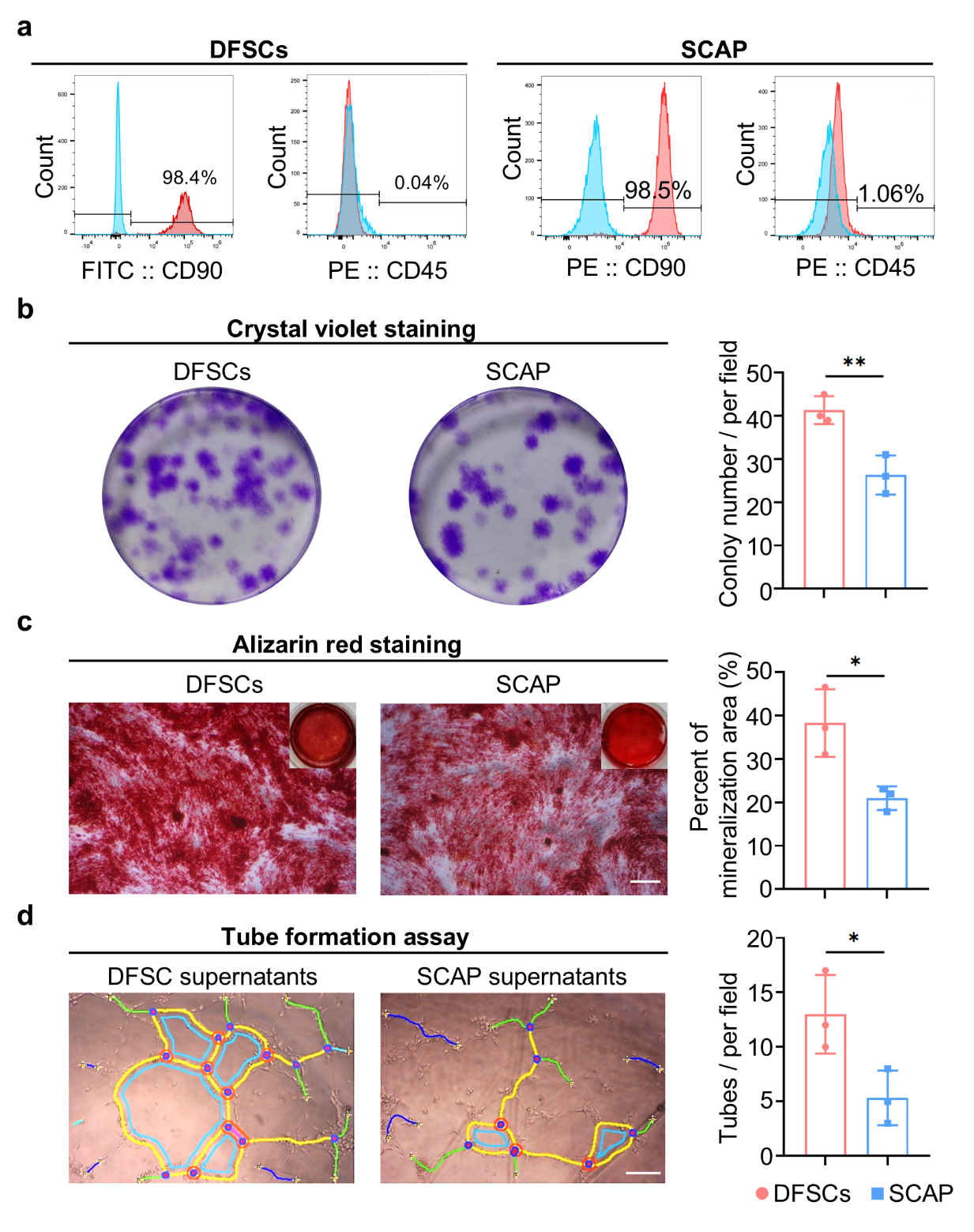
**

**Figure S4. Characterization and functional comparison between DFSCs and SCAP.** (a) Flow cytometric analysis for surface positive (CD90) and negative (CD45) markers. (b) Representative images of colonies formed by DFSCs and SCAP. (c) Alizarin red staining images showing osteogenic differentiation potential of DFSCs and SCAP. Scale bar = 100 μm. (d) Representative images showing tube formation of HUVECs treated by supernatants from conditioned medium of DFSCs and SCAP. Scale bar = 100 μm. n = 3 per group. **P* < 0.05; ***P* < 0.01.

**
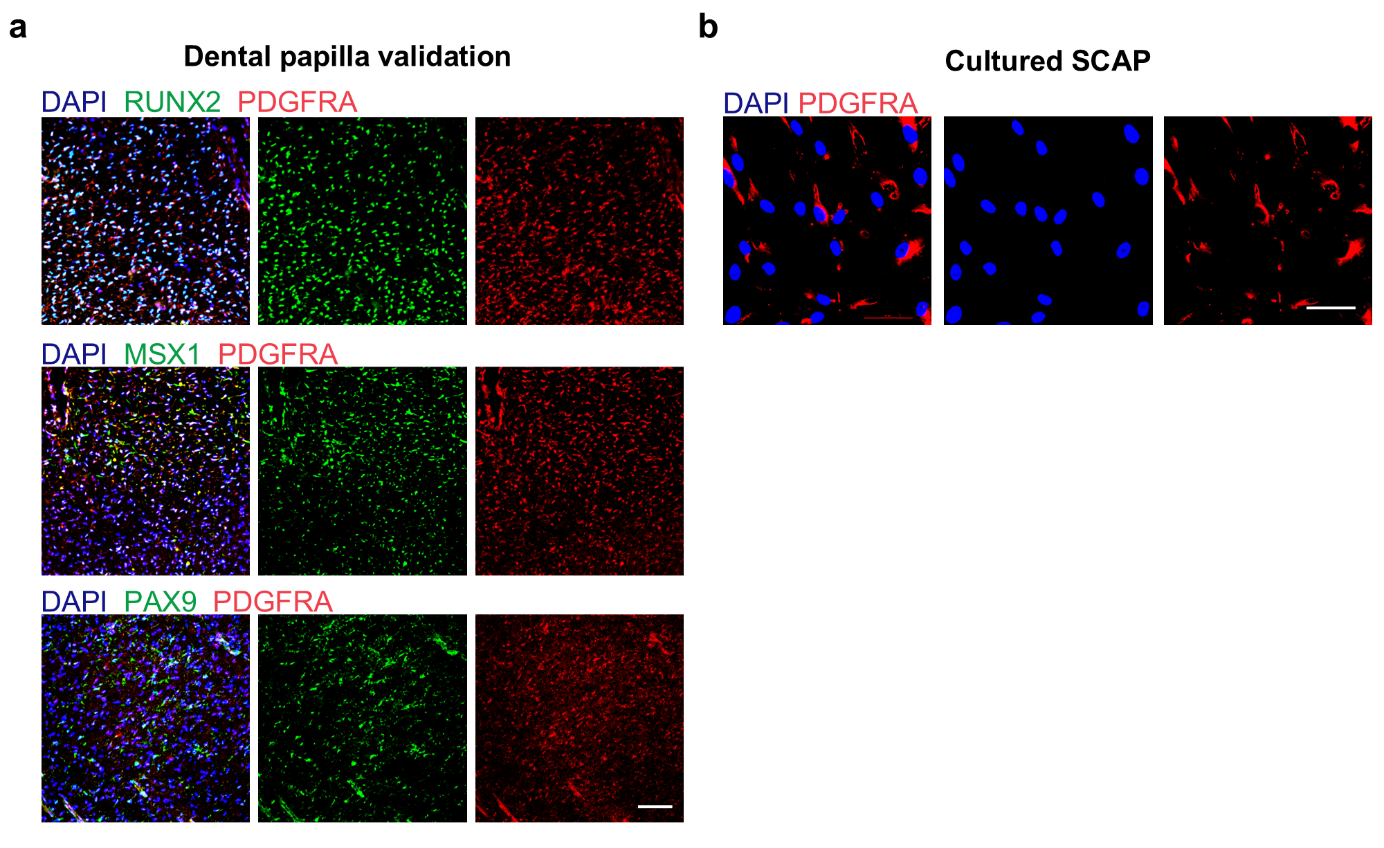
Figure S5. Validation of dental developmental genes in the dental papilla and cultured SCAP.** (a) IF staining images showing RUNX2 (green), MSX1 (green) or PAX9 (green) co-localized with PDGFRA (red) in the dental papilla tissue. Scale bar = 100 μm. (b) IF staining images showing PDGFRA^+^ (red) cells in cultured SCAP. Scale bar = 50 μm.


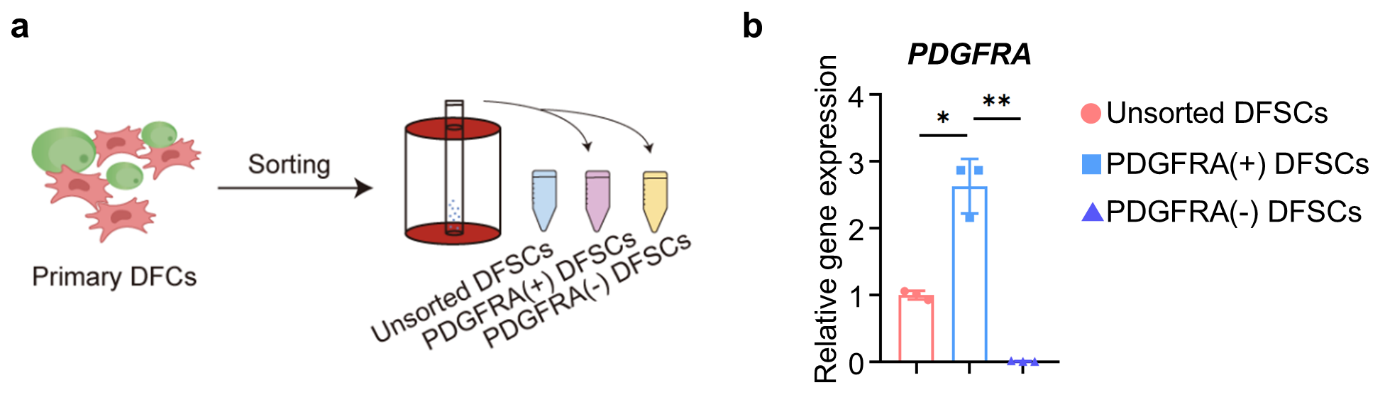


**Figure S6.** **Magnetic cell sorting and validation.** (a) Illustration of MACS for PDGFRA^+^ DFSCs. (b) Relative expression levels of *PDGFRA* by qRT-PCR in DFSCs after sorting. Data were presented as mean ± SD. n = 3 per group. **P* < 0.05; ***P* < 0.01.


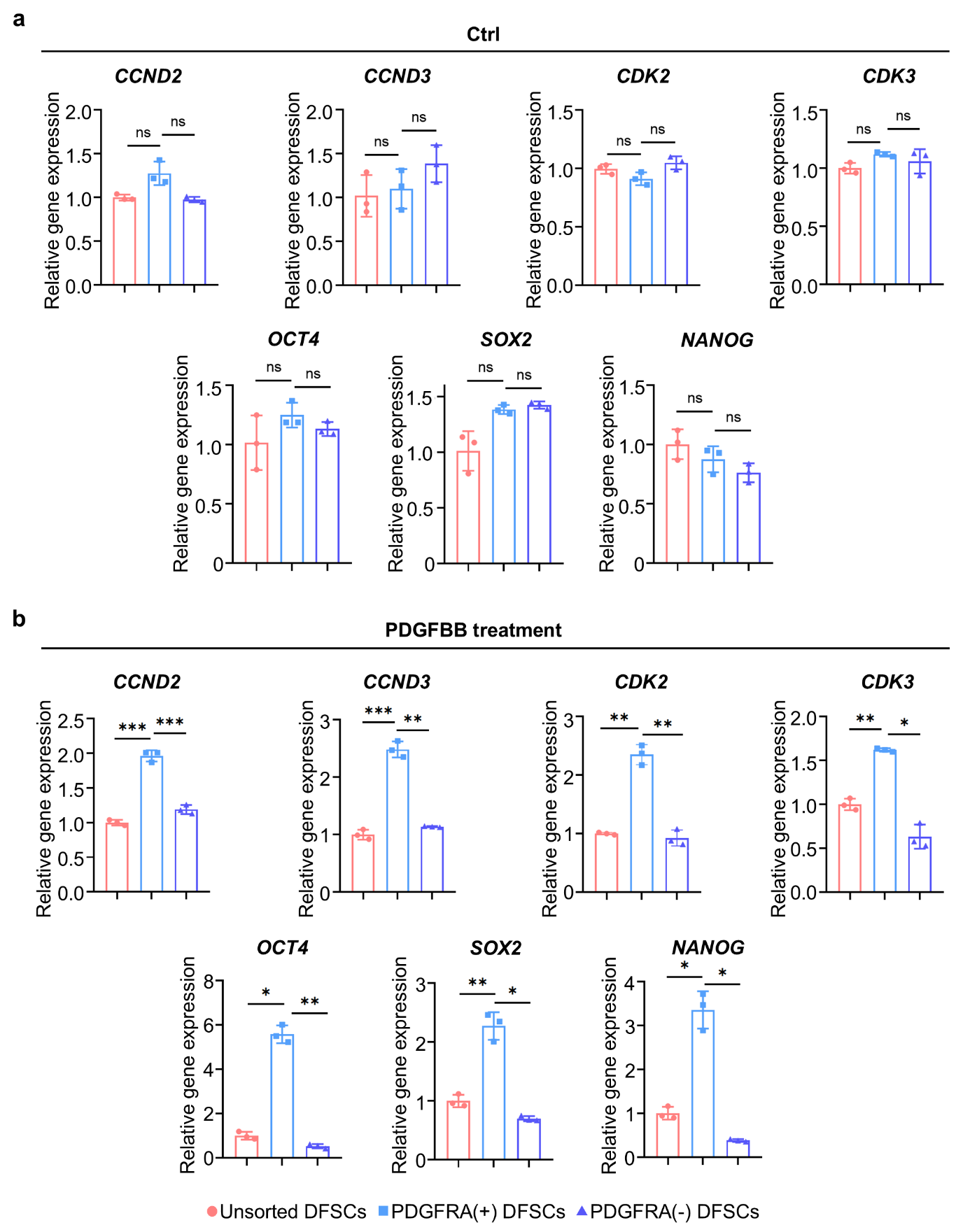


**Figure S7.** **Characterization for the cell cycle status and stemness of unsorted, PDGFRA^+^, and PDGFRA^-^ DFSCs.** (a) Relative expression levels of cell cycle-related genes and stem cell activity-related genes in DFSCs by qRT-PCR. (b) Relative expression levels of cell cycle-related genes and stem cell activity-related genes in DFSCs by qRT-PCR with PDGFBB treatment. Data were presented as mean ± SD. n = 3 per group. **P* < 0.05; ***P* < 0.01; ****P* < 0.001; ns, not significant (*P* > 0.05).


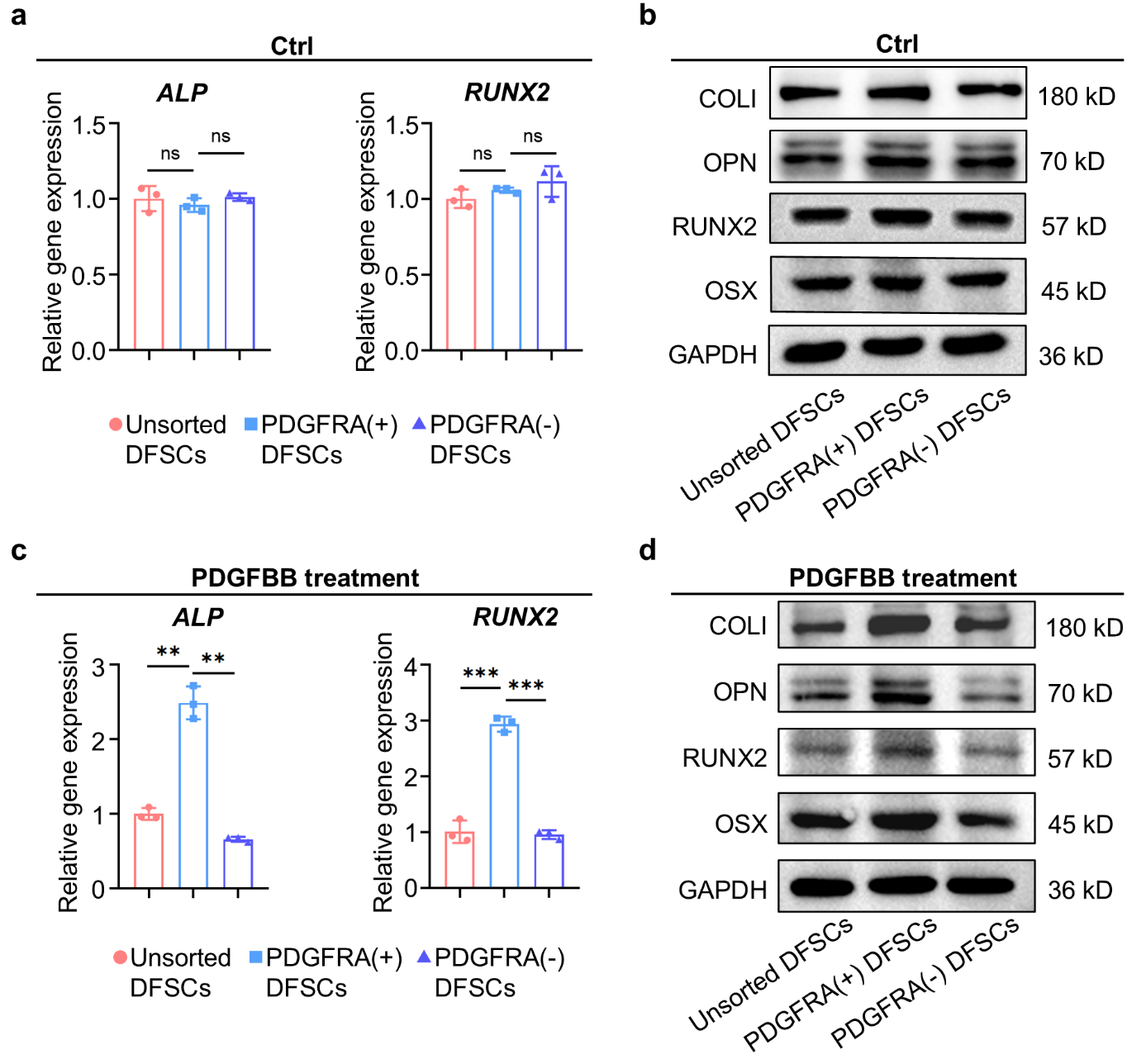


**Figure S8. Expression of osteogenesis-related genes and proteins in DFSCs.** (a) Relative expression levels of osteogenesis-related genes in DFSCs by qRT-PCR. (b) Western blot analysis of osteogenesis-related proteins in DFSCs. (c) Relative expression levels of osteogenesis-related genes in DFSCs with PDGFBB treatment by qRT-PCR. (d) Western blot analysis of osteogenesis-related proteins in DFSCs with PDGFBB treatment. Data were presented as mean ± SD. n = 3 per group. ***P* < 0.01; ****P* < 0.001; ns, not significant (*P* > 0.05).


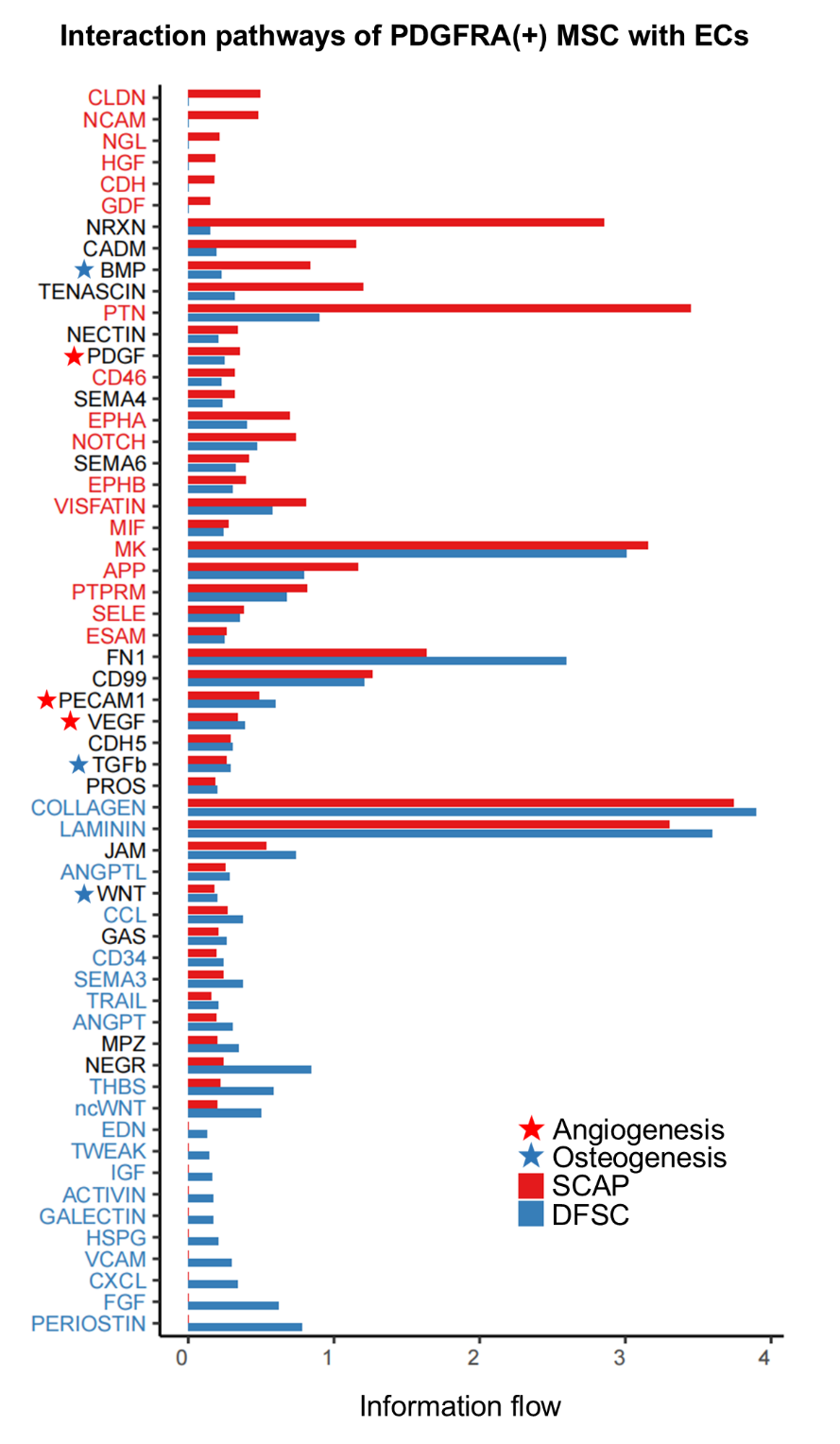


**Figure S9.** **Significant signaling pathways within inferred networks between PDGFRA^+^ MSCs and ECs.** The overall information flow of signaling networks is calculated by summarizing all communication probabilities. The length of columns represents the information flow calculated based on the sum of communication probabilities. The top signaling pathways colored red were enriched in PDGFRA^+^ SCAP, those colored blue were enriched in PDGFRA^+^ DFSCs, and those colored black were enriched in both PDGFRA^+^ SCAP and PDGFRA^+^ DFSCs.


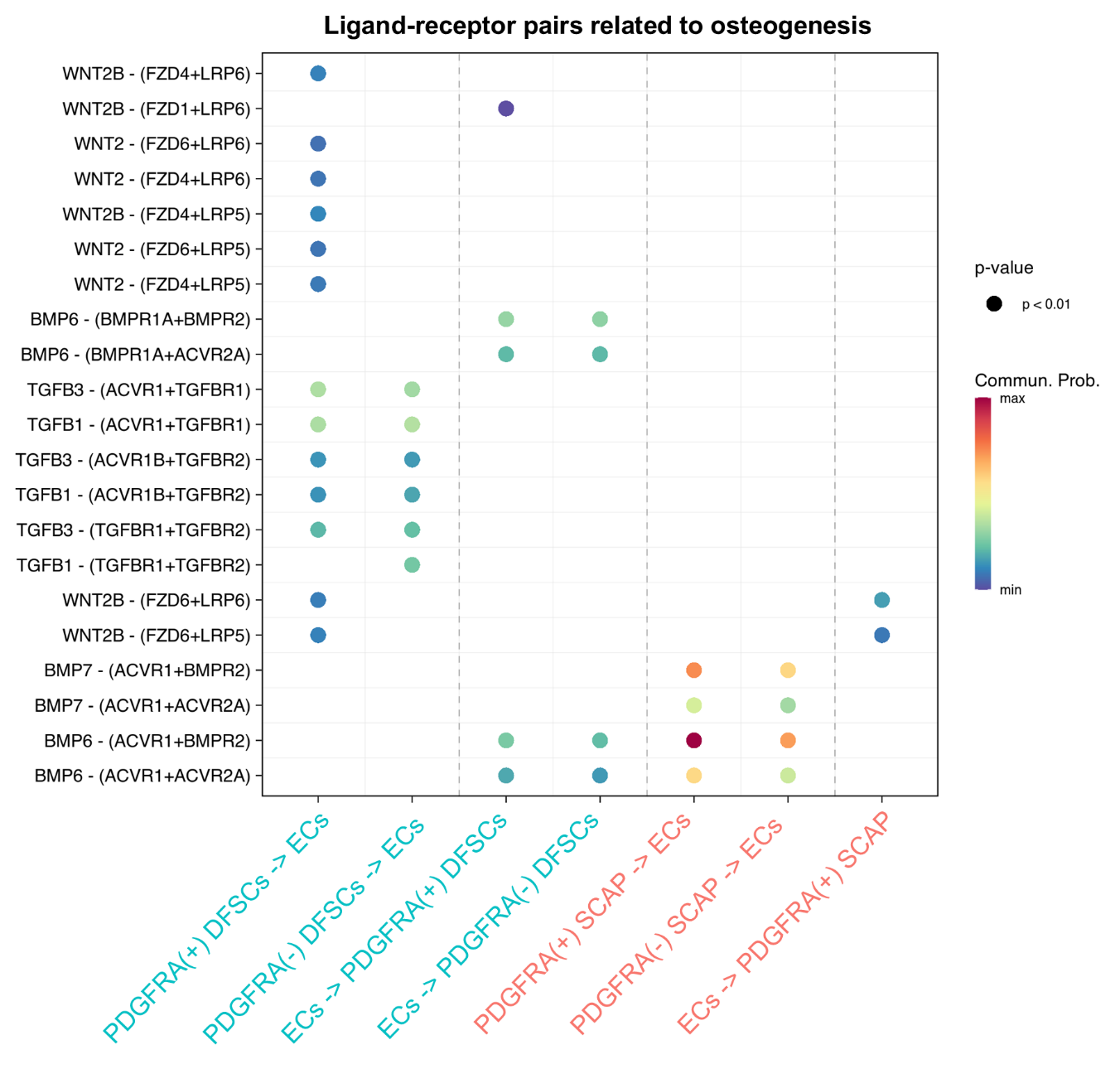


**Figure S10. Dot plot of significant ligand-receptor pairs related to osteogenesis.** Dots were colored by communication probabilities of ligand-receptor pairs.

**
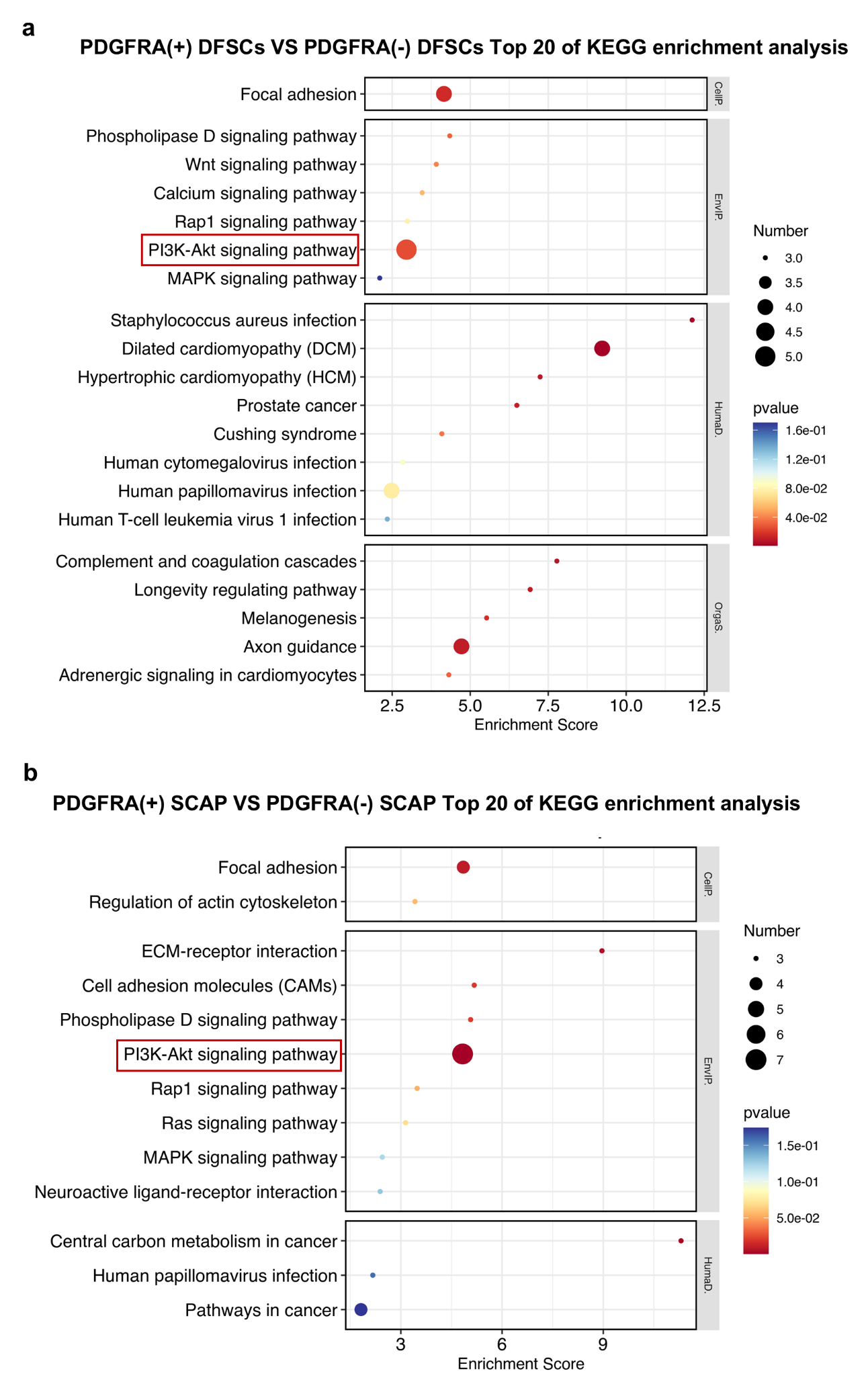
**

**Figure S11. KEGG enrichment analysis of DEGs between PDGFRA^+^ and PDFGRA^-^ MSCs.** (a) For DFSCs. (b) For SCAP.

**
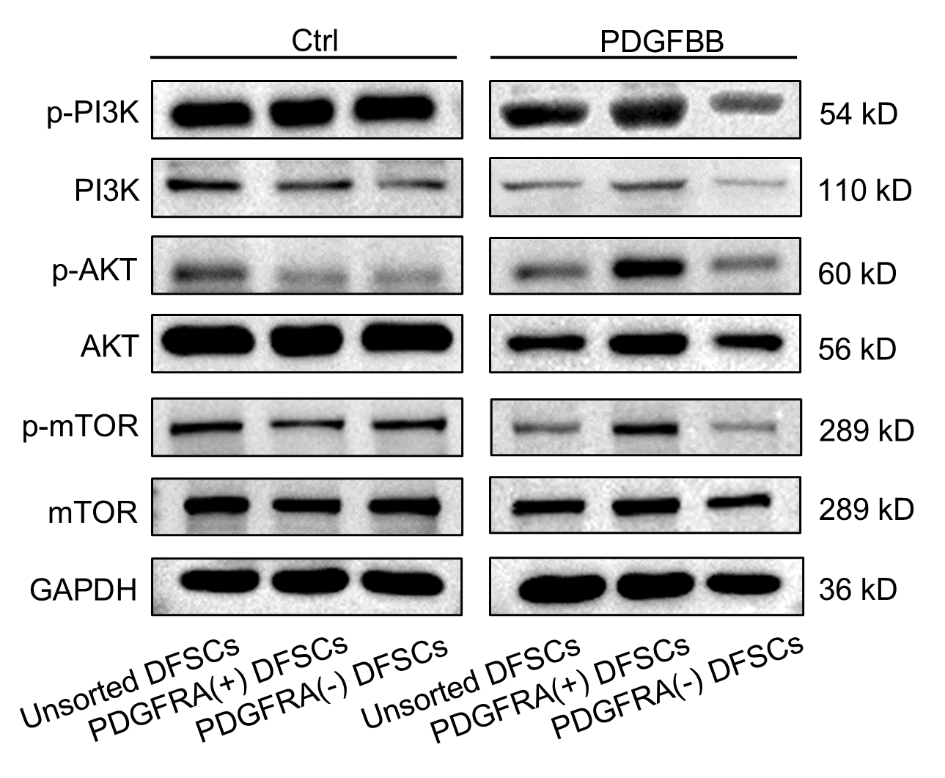
**

**Figure S12.** **Western blot analysis of PI3K/AKT/mTOR pathway proteins in DFSCs with and without PDGFBB treatment.**


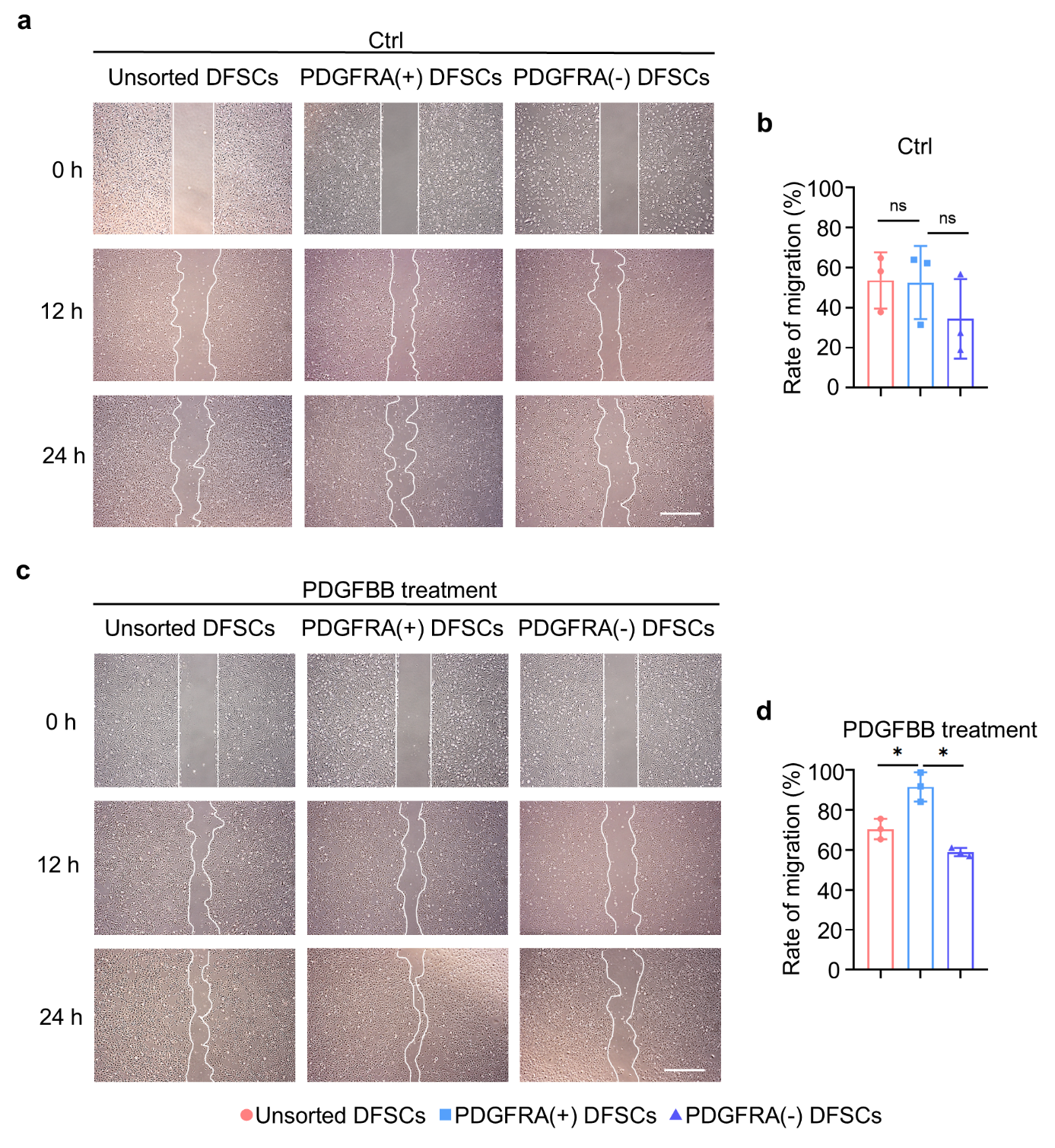


**Figure S13. The assessment of the paracrine potential of DFSCs to facilitate migration capacity of HUVECs.** (a) Representative scratch assay images showing migration of HUVECs treated by conditioned medium of DFSCs without PDGFBB. Scale bars = 500 μm. (b) Quantification of migration rate of HUVECs. (c) Representative scratch assay images showing migration of HUVECs treated by conditioned medium of DFSCs pretreated with PDGFBB. Scale bars = 500 μm. (d) Quantification of migration rate of HUVECs. Data were presented as mean ± SD. n = 3 per group. **P* < 0.05; ns, not significant (*P* > 0.05).


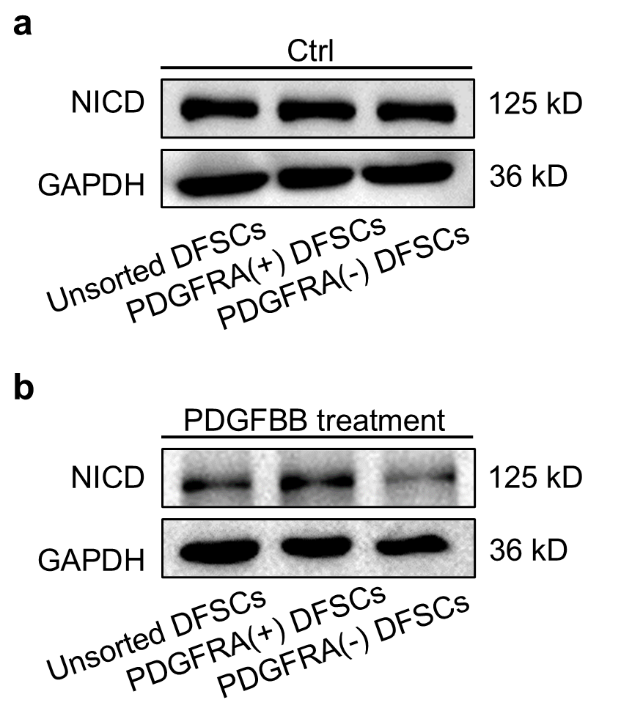


**Figure S14. Western blot analysis of NICD protein expression in HUVECs treated by conditioned medium of DFSCs. (a) For DFSCs pretreated without PDGFBB. (b) For DFSCs pretreated with PDGFBB.**


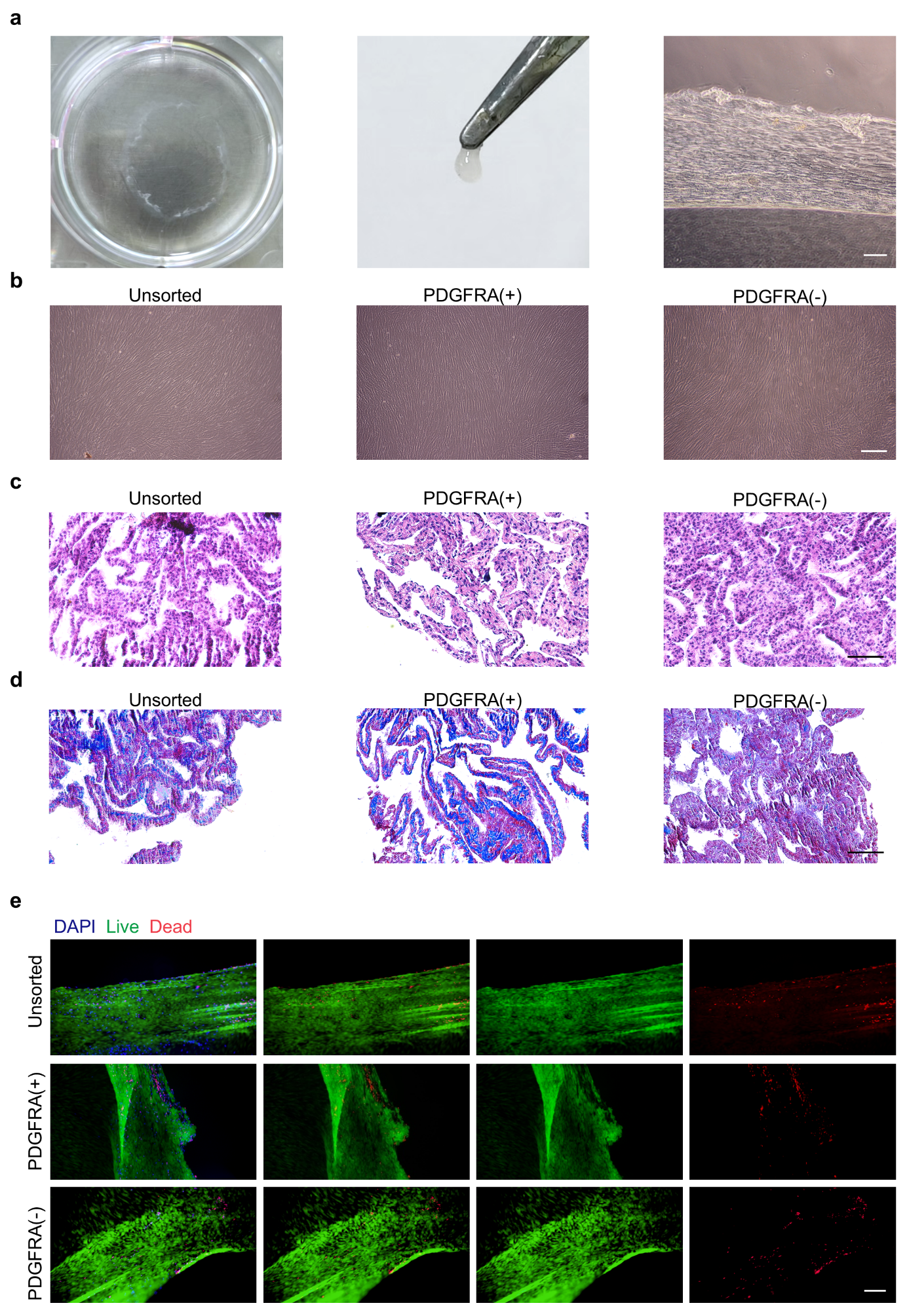


**Figure S15. Characteristics of DFSC aggregates.** (a) Macroscopic images of DFSC aggregates and the edge morphology under a microscope. Scale bar = 500 µm. (b) Microscopic images of DFSC aggregates. Scale bar = 500 µm. (c) H&E staining images showing continuous cell layers within the aggregates. Scale bar = 100 μm. (d) Masson’s trichrome staining images implying rich and compact ECM containing collagen deposited in the aggregates. Scale bar = 100 μm. (e) Live/dead staining of DFSC aggregates. Green: Calcein AM; Red: Propidium iodide; Blue: DAPI. Scale bar = 500 µm.


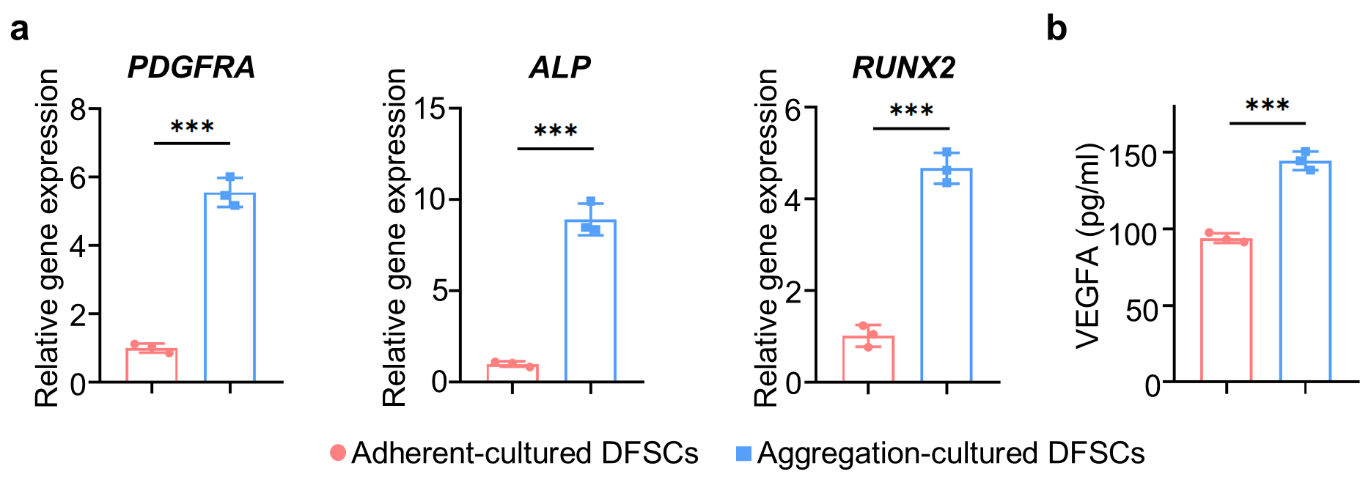


**Figure S16. Differences in cell function and molecular expression under adherent culture and cell aggregation conditions.** (a) Relative expression levels of *PDGFRA* and osteogenic-related genes including *ALP* and *RUNX2* in adherent-cultured DFSCs and aggregation-cultured DFSCs by qRT-PCR. (b) ELISA quantification of VEGFA concentrations in the conditioned medium from adherent-cultured DFSCs and aggregation-cultured DFSCs. Data were presented as mean ± SD. n = 3 per group. ****P* < 0.001.


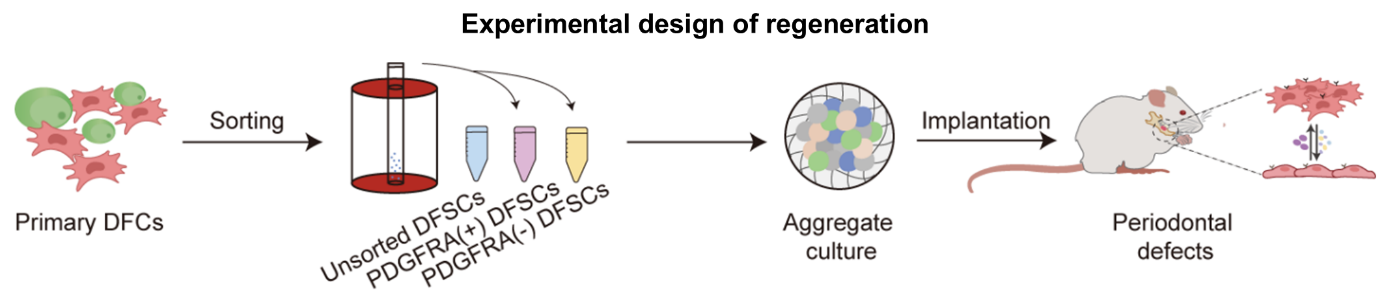


**Figure S17. The illustration for experimental design of periodontal bone regeneration using DFSC aggregates.**

**
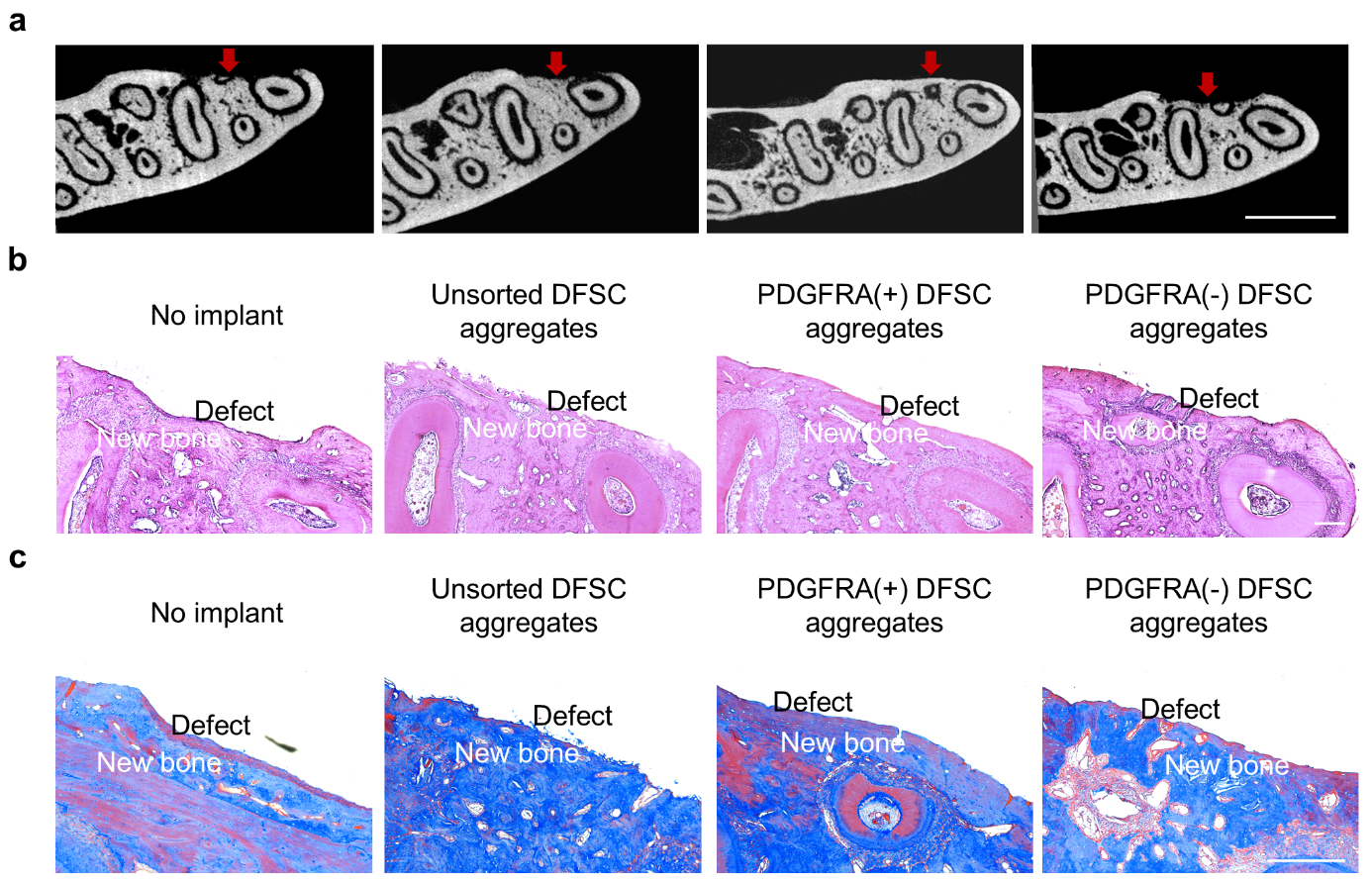
**

**Figure S18. Analysis of periodontal bone regeneration.** (a) Micro-CT images showing periodontal bone regeneration at 6 weeks after defect surgery and aggregate implantation in the original defect area indicated by red arrows. Scale bars = 2 mm. (b) H&E staining images showing the newly formed bone in the original defect area indicated by text annotation. Scale bar = 250 μm. (c) Masson’s staining images showing the newly formed bone in the original defect area indicated by text annotation. Scale bar = 250 μm.
