# Supplementary material for: Single-cell transcriptomics identifies PDGFRA+ progenitors orchestrating angiogenesis and periodontal tissue regeneration
Source: Int J Oral Sci. 2025 Jul 24;17:56. doi: 10.1038/s41368-025-00384-6 (PMC12289874; doi:10.1038/s41368-025-00384-6)

Original blot of Fig. 4d:
PDGFBB 27 kD

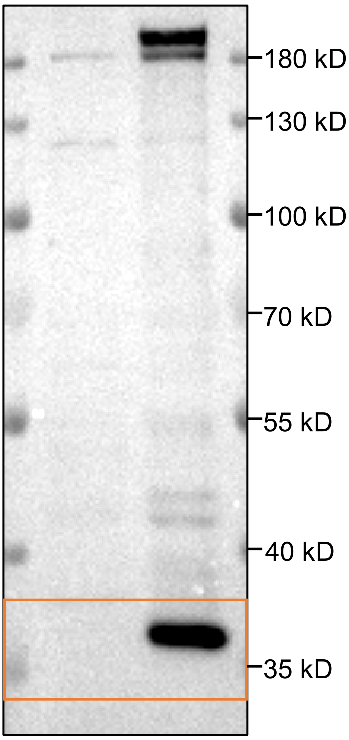

Original blot of Fig. 4d:
GAPDH 36 kD

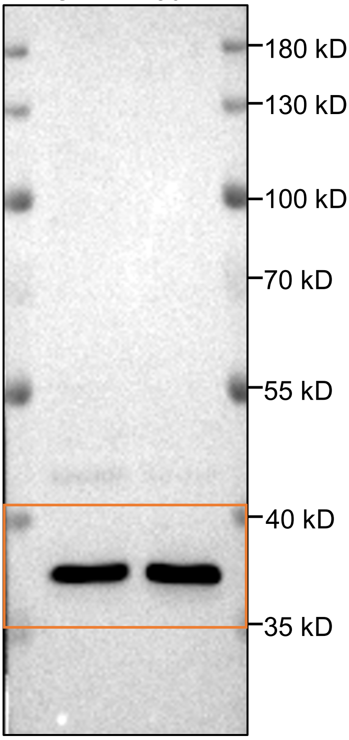

Original blot of Fig. S8b:
COLI 180 kD

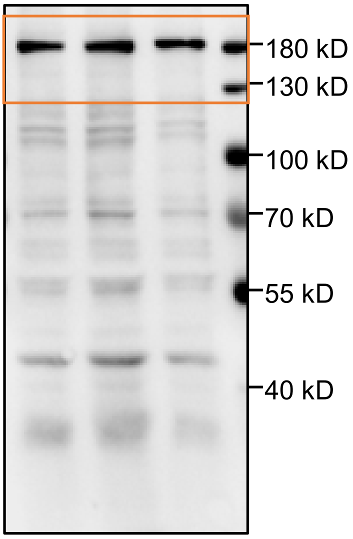

Original blot of Fig. S8b:
OPN 70 kD

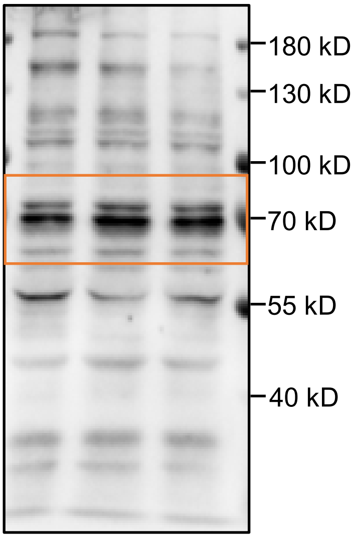

Original blot of Fig. S8b:
RUNX2 57 kD

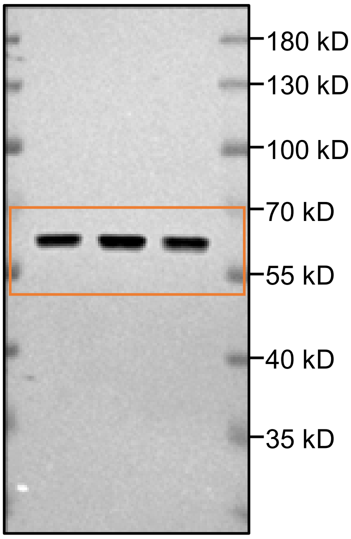

Original blot of Fig. S8b:
OSX1 45 kD

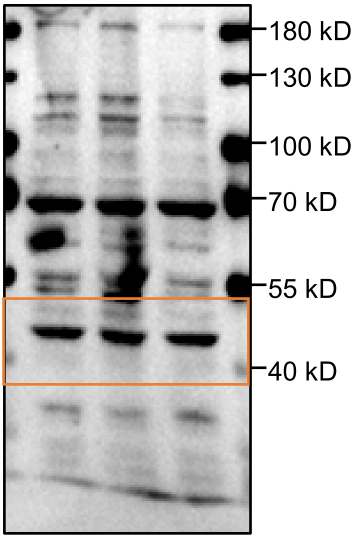

Original blot of Fig. S8b:
GAPDH 36 kD

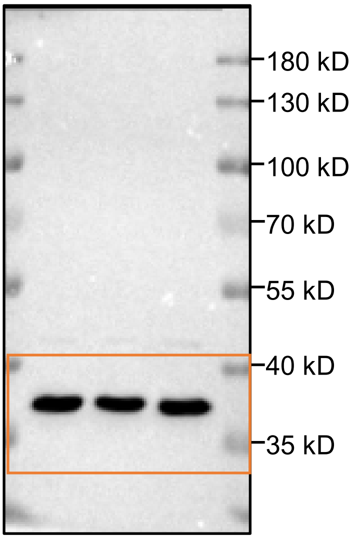

Original blot of Fig. S8d:
COLI 180 kD

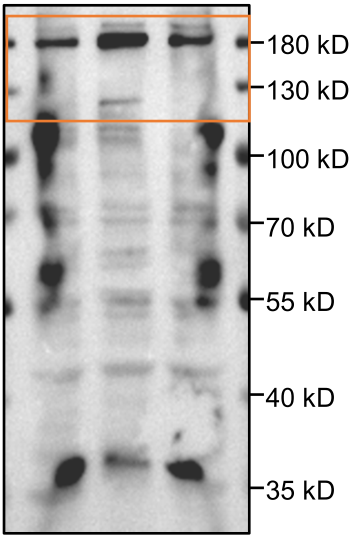

Original blot of Fig. S8d:
OPN 70 kD

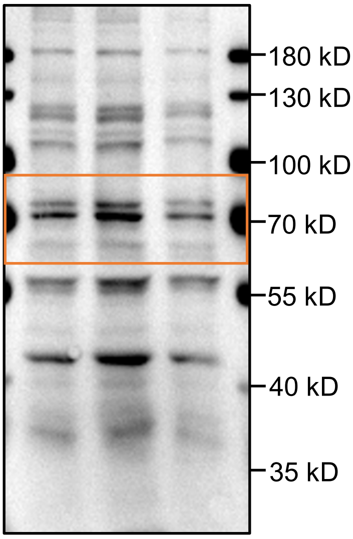

Original blot of Fig. S8d:
RUNX2 57 kD

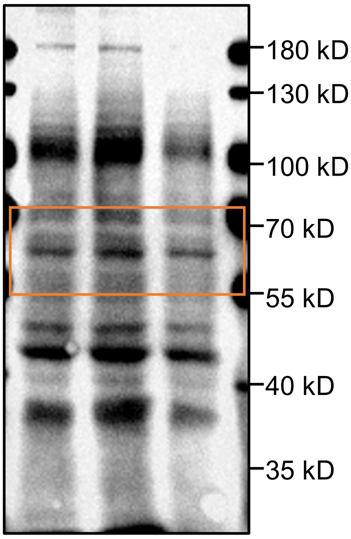

Original blot of Fig. S8d:
OSX1 45 kD

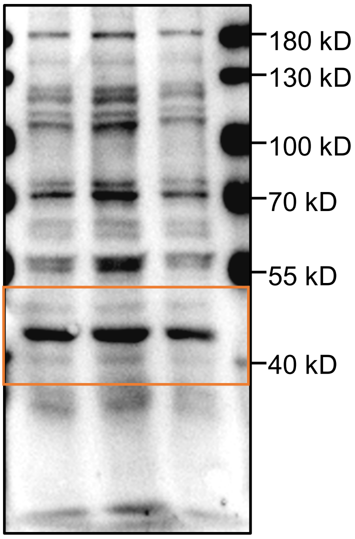

Original blot of Fig. S8d:
GAPDH 36 kD

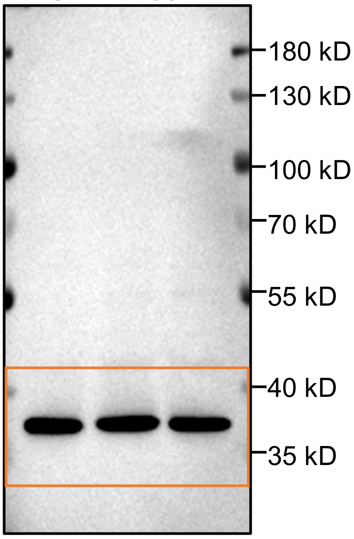

Original blot of Fig. S12:
p-PI3K 54 kD

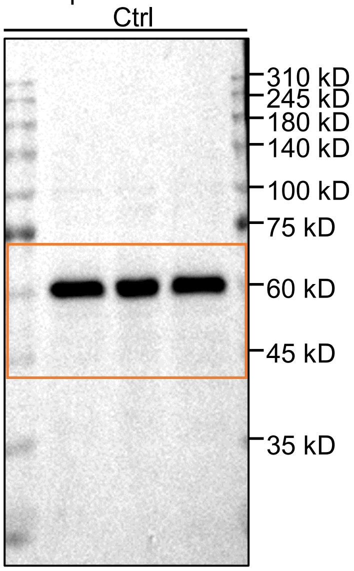

Original blot of Fig. S12:
PI3K 110 kD

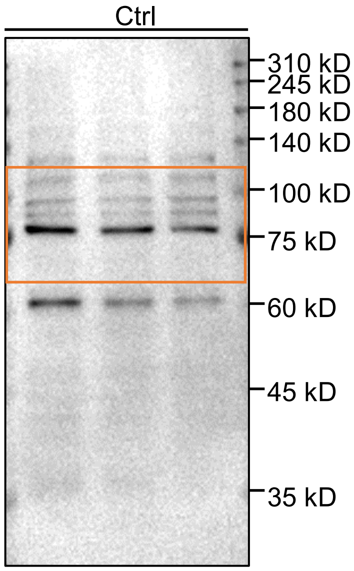

Original blot of Fig. S12:
p-AKT 60 kD

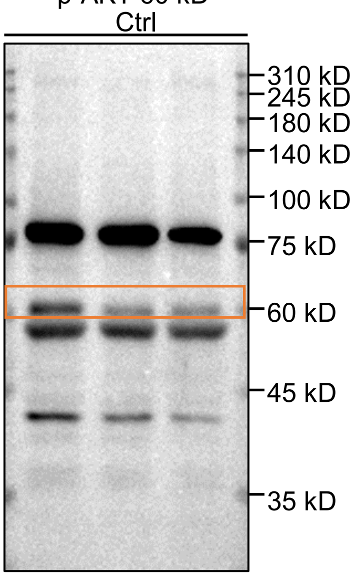

Original blot of Fig. S12:
AKT 56 kD

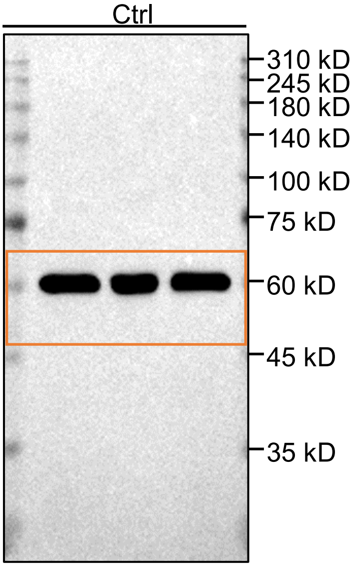

Original blot of Fig. S12:
p-mTOR 289 kD

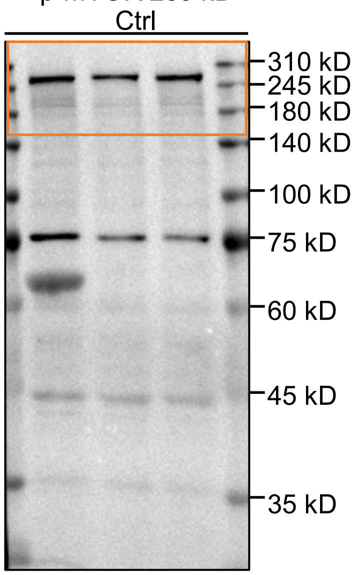

Original blot of Fig. S12:
mTOR 289 kD

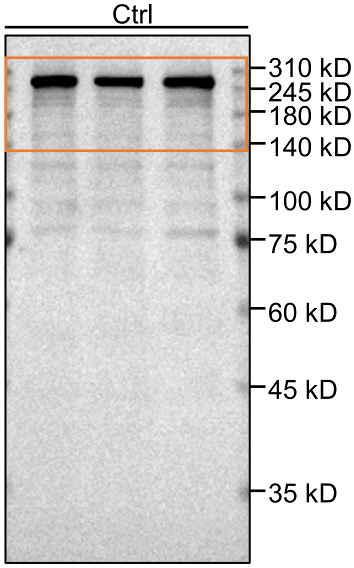

Original blot of Fig. S12:
GAPDH 36 kD

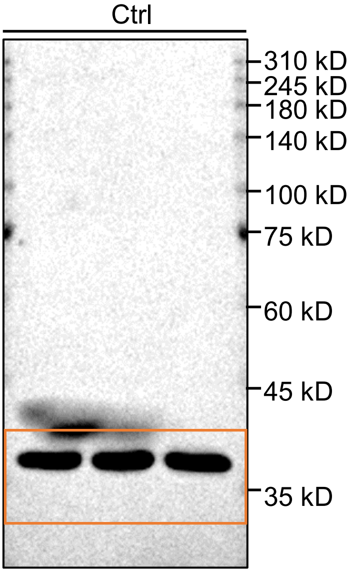

Original blot of Fig. S12:
p-PI3K 54 kD
PDGFBB

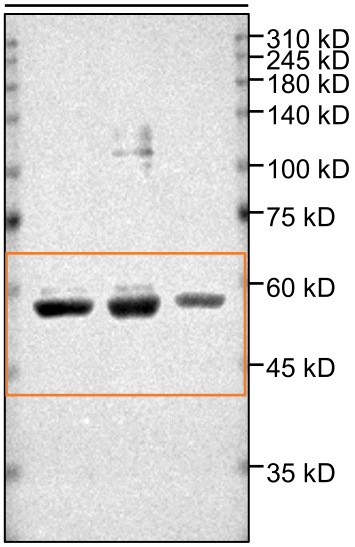

Original blot of Fig. S12:
PI3K 110 kD
PDGFBB

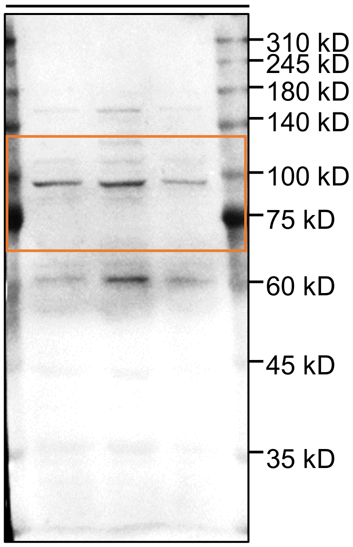

Original blot of Fig. S12:
p-AKT 60 kD
PDGFBB

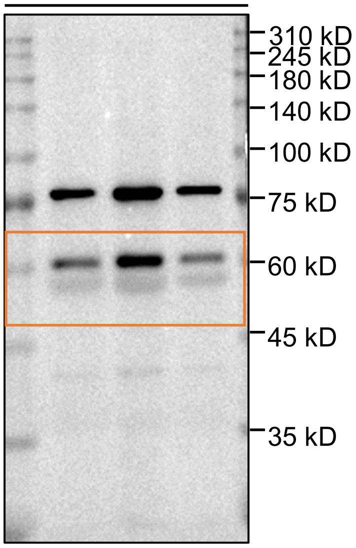

Original blot of Fig. S12:
AKT 56 kD
PDGFBB

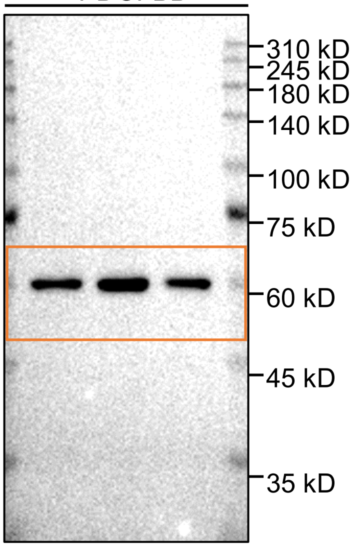

Original blot of Fig. S12:
p-mTOR 289 kD
PDGFBB

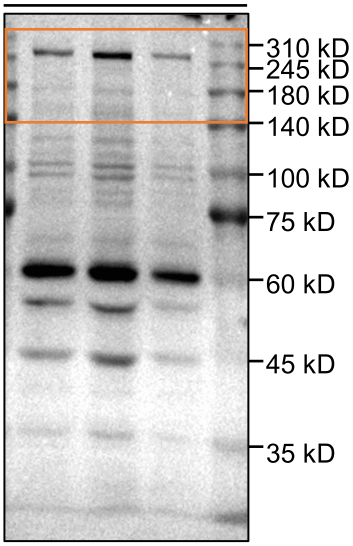

Original blot of Fig. S12:
mTOR 289 kD
PDGFBB

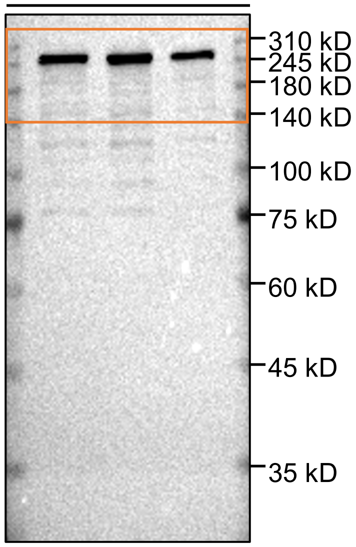

Original blot of Fig. S12:
GAPDH 36 kD
PDGFBB

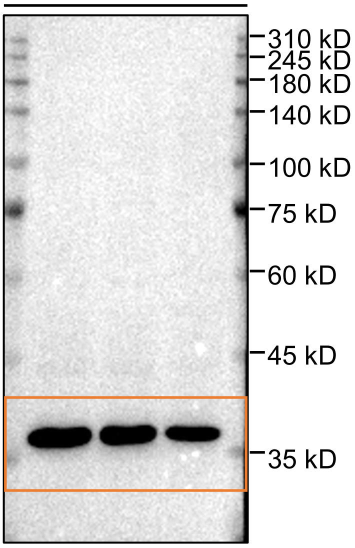

Original blot of Fig. S14a:
NICD 125 kD
Ctrl

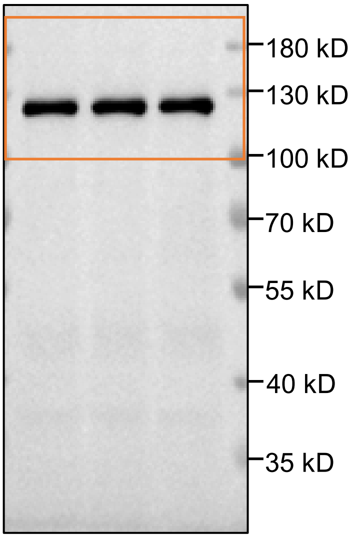

Original blot of Fig. S14a:
GAPDH 36 kD
Ctrl

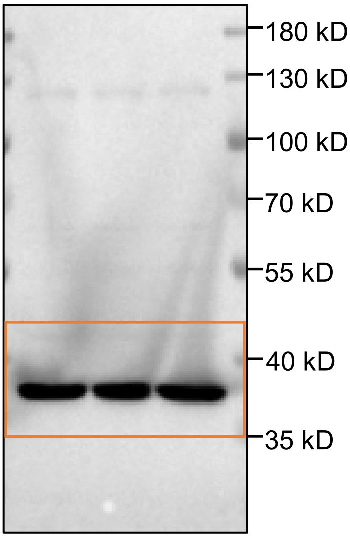

Original blot of Fig. S14b:
NICD 125 kD
PDGFBB

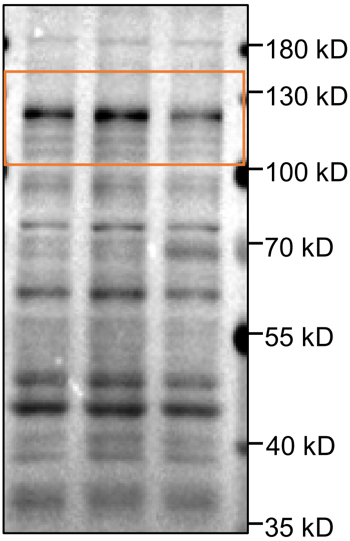

Original blot of Fig. S14b:
GAPDH 36 kD
PDGFBB

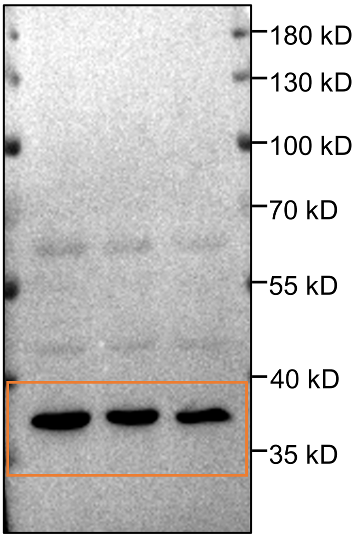

Supplement: Supplementary file 2 — Supplementary Information [file 41368_2025_384_MOESM2_ESM.pdf]
