# Supplementary figures and images for: Single-cell transcriptomics identifies PDGFRA+ progenitors orchestrating angiogenesis and periodontal tissue regeneration
Source: Int J Oral Sci. 2025 Jul 24;17:56. doi: 10.1038/s41368-025-00384-6 (PMC12289874; doi:10.1038/s41368-025-00384-6)

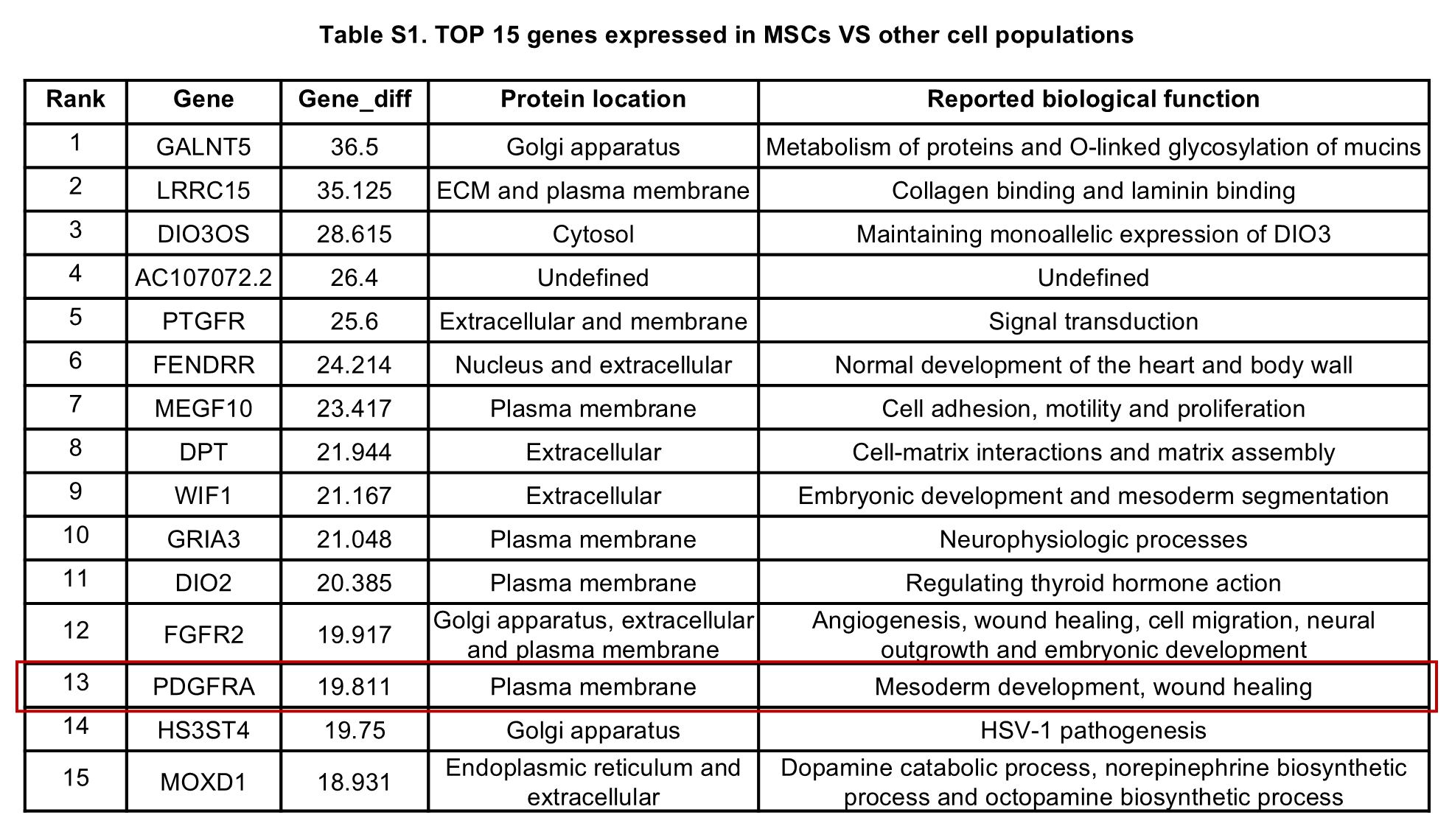

Supplement: Supplementary file 3 — Supplementary Table S1 [file 41368_2025_384_MOESM3_ESM.tif]
